# From burnout to behavior: the dark side of emotional intelligence on optimal functioning across three managerial levels

**DOI:** 10.3389/fpsyg.2024.1338691

**Published:** 2024-04-19

**Authors:** Samira A. Sariraei, Or Shkoler, Dimitris Giamos, Denis Chênevert, Christian Vandenberghe, Aharon Tziner, Cristinel Vasiliu

**Affiliations:** ^1^Department of Human Resources Management, HEC Montreal, Montreal, QC, Canada; ^2^Department of Psychology, Ariel University, Ariel, Israel; ^3^Tel-Hai Academic College, Kiryat Shmona, Israel; ^4^Peres Academic Center, Rehovot, Israel; ^5^Netanya Academic College, Netanya, Israel; ^6^Bucharest University of Economic Studies, Bucharest, Romania

**Keywords:** burnout, emotional intelligence, work motivation, organizational citizenship behaviors, work misbehaviors, managerial differences, optimal functioning, moderated mediation

## Abstract

**Introduction:**

Burnout has been typically addressed as an outcome and indicator of employee malfunctioning due to its profound effects on the organization, its members, and its profitability. Our study assesses its potential as a predictor, delving into how different sources of motivation—autonomous and controlled—act as mediational mechanisms in the association between burnout and behavioral dimensions of functioning (namely, organizational citizenship behaviors and work misbehaviors). Furthermore, the buffering effects of emotional intelligence across three different managerial levels were also examined.

**Methods:**

To this end, a total non-targeted sample of 840 Romanian managers (513 first-, 220 mid-, and 107 top-level managers) was obtained.

**Results:**

Burnout predicted motivation, which predicted work behaviors in a moderated-mediation framework. Contrary to our initial prediction, emotional intelligence augmented the negative association between burnout and motivation, exhibiting a dark side to this intelligence type. These findings are nuanced by the three managerial positions and shed light on the subtle differences across supervisory levels.

**Discussion:**

The current article suggests a relationship between multiple dimensions of optimal (mal)functioning and discusses valuable theoretical and practical insights, supporting future researchers and practitioners in designing burnout, motivation, and emotional intelligence interventions.

## Introduction

There are two sides to employee functioning. On the one hand, optimal functioning (“bright side”; Ryan and Deci, 2001, 2008; Gagné and Vansteenkiste, 2013; Van den Broeck et al., 2019) is manifested as the ideal worker template – with high well-being and positive work behaviors and attitudes. On the other hand, non-optimal or malfunctioning (“dark side”; Van den Broeck et al., 2008) is conceptualized as expressions of ill-being (e.g., burnout), work misbehaviors, and negative work attitudes. This typology is in line with the positive psychology literature suggesting that employees who function optimally can help their organizations become more productive and competitive by flourishing and fulfilling their potential at work (Van den Broeck et al., 2008; Trépanier et al., 2015; Aubouin-Bonnaventure et al., 2023). Most studies focus on the socio-contextual conditions that predict psychological (mal)functioning indexes (e.g., engagement vs. burnout; commitment vs. turnover intention; benevolent behavior vs. misbehavior) through motivational mechanisms (e.g., Schaufeli and Bakker, 2004; Van den Broeck et al., 2008; Fernet et al., 2012; Trépanier et al., 2015). However, researchers have either studied these indexes separately or as work outcomes with shared antecedents. The interplay between these work outcomes warrants further investigation. In other words, optimal (mal)functioning indicators have been mostly studied as “outcomes,” not as “predictors” of other work outcomes. It is unclear if and how dimensions of optimal functioning delineate motivational processes and affect other indexes of optimal functioning. In the current study, we intend to investigate whether burnout as an ill-being indicator of optimal functioning can predict behaviors through changes to individuals’ motivational sources. To elaborate, we investigate the impact that burnout, as an indicator of malfunctioning, might have on individuals’ work motivations, which in turn would affect work behaviors (i.e., behavioral dimension of optimal functioning).

The current article considers work burnout as the main malfunctioning indicator as it can be costly to organizations and/or their members. For example, workplace stress costs the U.S. economy more than $500 (USD) billion; each year, 550 million workdays are lost due to stress on the job (Moss, 2019). The World Health Organization (WHO) defines burnout as a syndrome resulting from chronic workplace stress that was not successfully managed. Specifically, many scholars define it as “a prolonged response to chronic emotional and interpersonal stressors” (e.g., Jackson and Maslach, 1982). As such, empirical findings support the notion that work burnout is a continuous process and show that individual burnout levels can change over time (Kristensen et al., 2005). However, most of the literature has examined burnout as an outcome or a mediator of adverse outcomes such as health problems (Toker et al., 2005; Ahola et al., 2014), low job performance (Maslach et al., 2001; Vahey et al., 2004), job dissatisfaction and withdrawal, absenteeism, and turnover (Leiter and Maslach, 2009; Laschinger, 2012; Rabatin et al., 2016), and low organizational commitment (Schaufeli and Enzmann, 1998; Maslach and Leiter, 2017). In the current article, we posit that because the experience of burnout occurs over time, it could elicit an implicit process leading to cognitive affliction, not only physiological diseases. In other words, we argue that burnout can be an important predictor of various consequences.

Moreover, the status of motivation in the literature as an important mediator predicting various indexes of optimal functioning has been empirically established (e.g., Self-Determination Theory [SDT]; Van den Broeck et al., 2008; Fernet et al., 2012; Trépanier et al., 2015; Deci et al., 2017). Capitalizing on SDT, the current paper treats motivation in the same way. However, contrary to the mainstream literature that treats optimal functioning indexes strictly as outcomes of motivational processes, we investigate a more nuanced interplay of these indexes, suggesting that they can simultaneously be predictors and outcomes of motivational processes.

**Figure 1:**
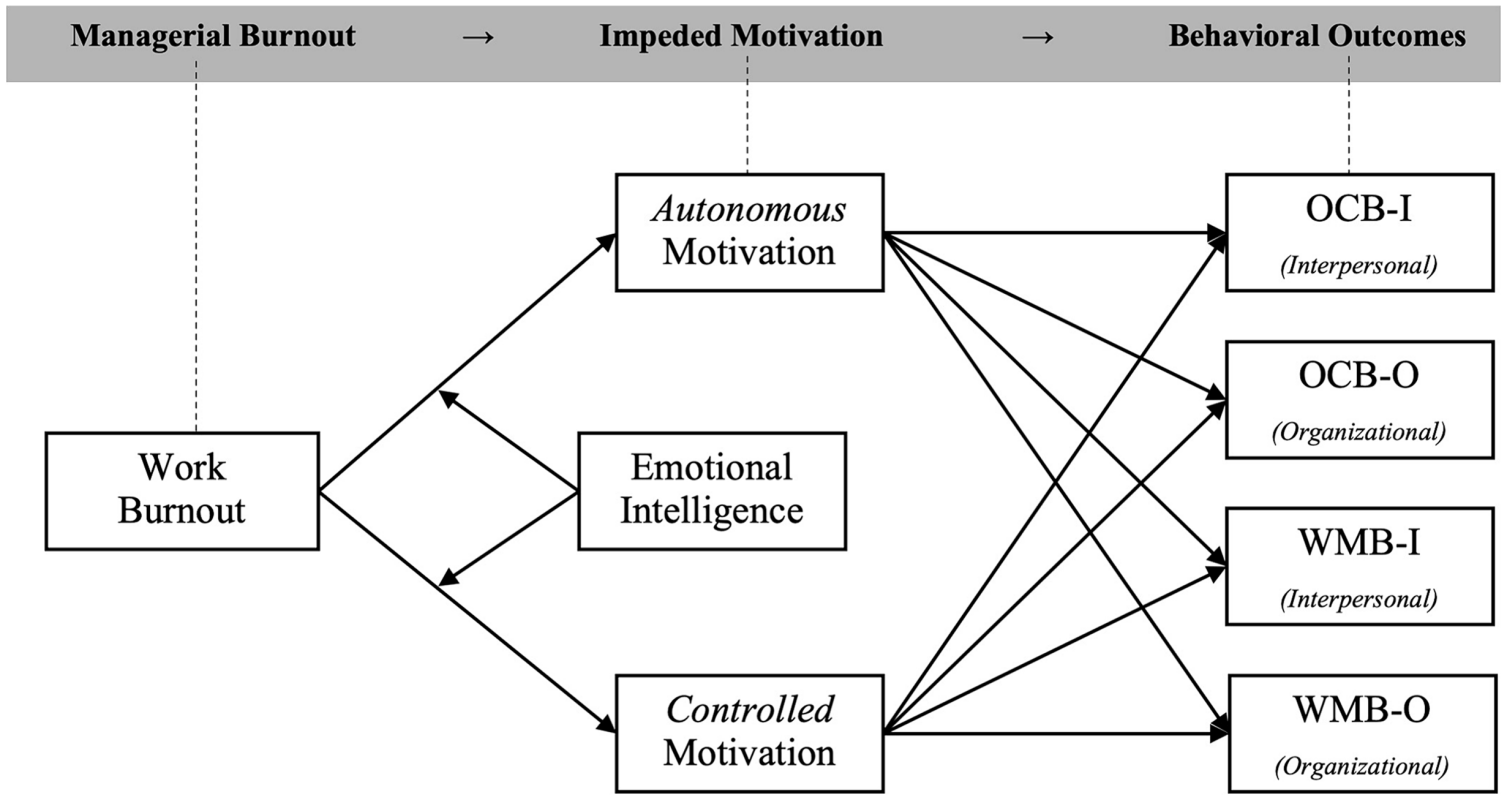
Overall research model for comparing junior managers (*n* = 513) medium managers (*n* = 220) and top managers (*n* = 107). OCB, organizational citizenship behaviors; WMB, work misbehaviors.

It is important to note that the literature on optimal functioning and SDT is central to our endeavor. It provides the canvas and the theoretical framework on which we drew our hypothesized model. These theories illustrate both the direction of association amongst the variables and the role (e.g., mediator) of each of them in the overall model (see Figure 1). However, to cement the various arguments for each specific hypothesis, other theories (e.g., Conservation of Resources theory, COR; Hobfoll, 1989; Emotional Intelligence theory; Boyatzis, 2001) are used to support the rationales.

Furthermore, the current research aims to investigate the buffering effect of emotional intelligence. According to Boyatzis (2009), “an emotional, intelligence competency is an ability to recognize, understand, and use emotional information about oneself that leads to or causes effective or superior performance” (p. 757). Emotional intelligence models share a common core of fundamental concepts. Emotional intelligence, in a general and parsimonious definition, refers to the ability to recognize and manage emotions in oneself and others and has four major components: Self-Awareness, Self-Management, Social Awareness, and Relationship Management. While terminology may evolve with theoretical advancements, these domains remain consistent across various EI theories. It is broadly conceptualized as an ability or competency to identify, monitor, and manipulate one’s and others’ emotions to varying degrees and generally has a positive mindset around it (Boyatzis, 2001, 2009). Additionally, it is widely agreed that emotional intelligence can be developed and enhanced at the individual level (e.g., Boyatzis, 2001) like self-efficacy, and it is also perceived to be predominantly positive. Thus, we were interested in its intricate moderating effects (e.g., Shkoler and Tziner, 2017) for both theoretical and practical reasons.

Additionally, the research model (Figure 1) was tested and compared across three managerial levels (first-level, mid-level, and top-level managers). First-level managers, also known as junior or office managers, supervise employees with non-managerial roles. Mid-level managers, also known as department managers, are responsible for specific departments or functional areas within the organization, like marketing or operations, and can manage first-level managers. Top-level managers, or executive managers, hold senior leadership positions and can manage mid-level managers. They oversee setting the overall strategic direction and ensuring the organization’s success. These different positions are associated with different workloads, resources, needs, political sway, expectations, demands, challenges, and other characteristics, which justifies the exploration of potential differences across managerial positions (e.g., Delaye and Boudrandi, 2010; Lundqvist et al., 2013; Chullen, 2014; Rabenu et al., 2019; Samson et al., 2021).

Moreover, a Romanian sample of managers has been targeted in this study. In choosing Romania as the cultural context for our study, several factors were considered, including its membership to the European Union (EU) and its unique sociocultural context.

First, Romania’s integration into the EU entails the adoption of key elements of labor legislation at the European level (Jordan et al., 2021) bringing it under the purview of directives such as the Framework Directive 89/391 on Occupational Safety and Health (OSH) and the Directive on the Organization of Working Time 88/2003. These directives set forth regulations aimed at safeguarding the well-being of employees, including provisions on workload, limits of working time, and provisions for rest, all of which are directly relevant to the study of burnout. However, despite efforts to align with EU standards, discrepancies exist, leading to persistence of issues such as overtime work, task overload, and demanding work conditions (Adăscăliței and Guga, 2018).

Furthermore, Romania presents a unique cultural and economic context that enriches our study’s implications. As a country in transition with the entry of large multinational companies into its market, Romania grapples with a blend of traditional values and modernization efforts, which can influence workplace dynamics with an infusion of diverse managerial approaches and motivations. Therefore, while Romania benefits from the presence of multinational corporations, regulations aimed at safeguarding the well-being of employees, and diverse managerial approaches, it also faces labor-related challenges that warrant investigation of motivation and optimal functioning among these employees.

### Goals and contributions

The current study has four main goals: (1) to examine burnout’s role as a predictor, not an outcome/mediator, and to establish its adverse effects on work behaviors; (2) to investigate the mediational mechanism of work motivation (autonomous vs. controlled); (3) to gauge the extent to which emotional intelligence conditions the association between burnout and work motivation; and (4) to compare our model across three managerial levels.

By achieving these goals, the current study contributes to the literature in multiple ways. First, to the best of our knowledge, the current study is pioneering the examination of burnout as a predictor of work context in the I/O-Psych and OB literature. We follow examples such as Lonsdale and Hodge (2011) and Martinent et al. (2014), where burnout predicted athlete motivation, bringing the notion to the OB domain. By doing so, the current study also contributes to the literature on optimal functioning by investigating whether negative well-being (i.e., burnout) influences the dark and bright aspects of the behavioral dimensions of functioning. Second, as emotional intelligence has a profound impact on work-related outcomes (e.g., Shkoler and Tziner, 2017), it would be an intriguing endeavor to include it in the model for theoretical and practical reasons. Third, the multi-group comparison across three managerial levels should shed light on the differences amongst various managerial job categories that may help revisit organizational practices related to these positions and how to optimize their associated attitudes and behaviors.

## Theoretical background

### Optimal functioning and work motivation

“Optimal functioning” is defined as the “manifestation of intra- and interpersonal growth and development in terms of employee well-being (e.g., positive emotions, vitality), attitudes (e.g., job satisfaction, organizational commitment), and behavior (e.g., performance, proactivity, and collaborative behaviors)” (Van den Broeck et al., 2019, p. 21). Despite conceptual differences surrounding the term, there is consensus on its multidimensionality (Van den Broeck et al., 2008). In addition, the occupational health psychology literature identified three main dimensions of optimal functioning: (1) psychological (e.g., a state of well-being vs. ill-being) (2) attitudinal (e.g., commitment vs. turnover intention), and (3) behavioral (e.g., in-role performance) (e.g., Dagenais-Desmarais et al., 2014; Trépanier et al., 2015, 2023; Fernet et al., 2016; Gradito Dubord and Forest, 2023; Vanbelle et al., 2023). These notions have profound effects on motivation of individuals (e.g., Shkoler and Rabenu, 2023; Shkoler and Tziner, 2022) as will be elaborated in the next sections. To better understand and contextualize these complex notions, this paper capitalizes on the SDT approach to human motivation, by which we want to shed light on motivational mechanisms as pathways that link optimal functioning to its behavioral outcomes.

SDT (Ryan and Deci, 2000, 2001) has developed into a grand paradigm of human motivation that specifies the conditions stimulating optimal functioning (bright side) or, otherwise, eliciting malfunctioning (dark side) (Van den Broeck et al., 2008). According to SDT, individuals are inherently inclined towards growth (Ryan and Deci, 2000) and employees are likely to function optimally in an environment where this tendency is cherished and encouraged (Van den Broeck et al., 2008; Trépanier et al., 2015). Conversely, if the work environment impedes the individual’s growth, their vulnerabilities often result in dysfunction (Van den Broeck et al., 2008). There is abundant literature on the contextual and intrapersonal factors that promote optimal functioning or hinder malfunctioning (Baard et al., 2004; Lynch et al., 2005; Gagné and Deci, 2005; Van den Broeck et al., 2008; Vansteenkiste, et al., 2010; Deci et al., 2017). Nevertheless, the literature about the effects of different dimensions of optimal functioning is still scarce. Studies investigating the “happy-productive worker thesis” have explored the relationship between well-being and performance, showing support for well-being as a positive determinant of productivity (Peiró et al., 2019; Isham et al., 2021; Aubouin-Bonnaventure et al., 2023).

Furthermore, work motivation is often defined as “a set of energetic forces that originates both within as well as beyond an individual’s being, to initiate work-related behavior, and to determine its form, direction, intensity and duration” (Pinder, 1998, p. 11). Motivation animates individuals to persist in courses of action until the acts are completed (Pinder, 2014) and is studied through processes by which an individual’s internal psychological forces – in conjunction with external, environmental, or contextual forces – determine the direction, intensity, and persistence of personal behavior aimed at goal attainment (Kanfer et al., 2017; Tziner et al., 2020b). The most prominent paradigm to discuss motivation is SDT (Ryan and Deci, 2000), which posits that individual differences and contextual characteristics of a job are related to motivation and work outcomes by satisfying vs. frustrating the three basic needs of autonomy, competence, and relatedness (Vansteenkiste et al., 2010, 2020; Deci et al., 2017), such as working climate (Tziner and Shkoler, 2018; Shkoler et al., 2021a,b).

According to SDT (Deci and Ryan, 1980), people are intrinsically motivated when they engage in an activity because they enjoy it and have a sense of fulfillment from doing it. However, in the case of extrinsic motivation, satisfaction is not derived from the activity itself but rather from the external consequences to which it leads, such as tangible or verbal rewards (Gagné and Deci, 2005; Deci et al., 2017). Typically, work motivation, under the SDT framework, is categorized into six different subtypes, from the least motivated (i.e., amotivation) to the most intrinsically motivated (i.e., intrinsic motivation) ones. Between these two extremes are the remaining four sub-types of extrinsic motivations that vary in the extent to which their regulation is autonomous vs. controlled: (A) *externally regulated* behavior (i.e., externally regulated behavior occurs to satisfy an external demand or reward contingency) (B) *introjected regulation* (i.e., an internalized form of contingency, involving the protection of self-worth, avoiding feelings of guilt or anxiety, or gaining others’ respect) (C) *identified regulation* (i.e., conscious valuing of the action and its intended consequences), and (D) *integrated regulation* (i.e., full integration of values guiding the behavior and values defining one’s self-concept). It is important to note, however, that since the focus of the current paper is not on the individual sub-types, we grouped them into the higher-order classification of autonomous vs. controlled motivation.

The autonomous/controlled typology of motivation arises from grouping these forms of behavior regulation into two distinct groups. Identification, integration, and intrinsic motivation are the prototypes of self-determined (*autonomous*) motivation, while amotivation, external regulation, and introjection are categorized as non-self-determined (*controlled*) motivation (Ryan and Deci, 2000; Gagné and Deci, 2005; Tremblay et al., 2009; Deci et al., 2017). SDT proposes that more autonomous forms of motivation are associated with increased scores of “optimal functioning” indexes (i.e., well-being, work attitudes, and behavior; Ryan and Deci, 2000; Van den Broeck et al., 2008; Fernet et al., 2012; Trépanier et al., 2015; see Van den Broeck et al., 2021 for a recent meta-analysis), indicating that the beneficial effects of autonomous motivation go beyond employees’ well-being. Interestingly, Ryan and Deci (2008) contend that controlled forms of self-regulation are energy-consuming while autonomous forms of self-regulation are not.

As mentioned above, the associations between multiple indicators of optimal functioning with each other have yet to be studied under the SDT umbrella. However, as SDT theorizes motivation as the *process* explaining the effects of predictors of optimal functioning (Gagné and Deci, 2005; Deci and Ryan, 2008; Liu et al., 2011), some researchers have examined the role of well-being on motivation. In the work context, Dagenais-Desmarais et al. (2018) examined the longitudinal relationship of psychological health (as indexed by well-being) to motivation. Based on panel data, their goals were to test (1) standard causality (motivation types predict well-being) (2) reverse causality (well-being predicts motivation), and (3) reciprocal causality (both motivation and well-being at work affect each other). The study found partial support for standard causality. However, they also found that well-being at work predicted autonomous and controlled motivation 6 months later. Earlier empirical support for the predictive role of psychological health on motivation was reported by Isen and Reeve (2005) and demonstrated that positive affect, in general, leads to increased intrinsic motivation. In sum, studies scrutinizing the predictive role of dimensions of optimal functioning are limited and the existing literature mostly focuses on well-being and performance. The predictive role of burnout as a manifestation of malfunctioning on behavioral indicators of optimal functioning (e.g., benevolent behavior) and malfunctioning (e.g., work misbehavior) are rather overlooked.

Next, we review the literature on burnout, organizational citizenship behaviors (OCB), and work misbehaviors (WMB), and their relationship with work motivation.

### Burnout

Burnout has been a pressing topic for researchers and practitioners for decades because of its adverse impact on people’s personal and professional lives and its prevalence among workers (Maslach and Jackson, 1981; Cordes and Dougherty, 1993). Academic research and health organizations report that job burnout is consistently increasing, and our potential to ameliorate and protect workers against burnout is limited. For instance, interventions designed to prevent or reduce burnout have not been effective in diverse industries (Ahola et al., 2017; Dreison and Lagges, 2017; Dreison et al., 2018; De Simone et al., 2021). Since burnout is no longer considered as an isolated factor, but rather a reality rooted in organizations and society, understanding the means to mitigate its effects is today a priority.

Burnout is “a prolonged response to chronic emotional and interpersonal stressors on the job and is defined by the three dimensions of emotional exhaustion, depersonalization, and reduced personal accomplishment (Maslach et al., 2001, p. 397). *Emotional exhaustion* is characterized by feeling emotionally drained and exhausted by one’s work (Maslach et al., 2001). *Depersonalization* or *cynicism* “refers to a negative, callous, or excessively detached response to various aspects of the job” (Maslach et al., 2001, p. 399). This aspect is particularly crucial when considering the role of managers; if they are insensitive or outright abusive, this can have a significant negative impact on their subordinates (Tepper, 2007) and the organization at large. *Reduced personal accomplishment* refers to the feeling of decreased competence or achievement in one’s work. Individuals experiencing reduced personal accomplishment may feel that their work is not meaningful or that they are not making a difference. Burnt-out employees tend to exhibit a noticeable reduction in the sense of personal accomplishment, often viewed as the result of the previous two dimensions.

Research shows that without definitive changes in work settings, burnout can remain constant for extended periods (Maslach et al., 2001; Maslach and Leiter, 2017). Qualitative (e.g., Maslach, 1982) and quantitative longitudinal (e.g., Bakker et al., 2000; Taris et al., 2005; Leiter and Maslach, 2009; Mäkikangas et al., 2021) studies have provided support for the sequence of stages in burnout such that first, people would experience a demanding workload that taxes their emotional resources, and so they experience emotional exhaustion. To cope with this overload, people would detach themselves from their work, develop adverse reactions to the job, and treat people in callous and cynical ways (depersonalization). Over time, people would question their ability to do the job well and experience feelings of inadequacy and failure or reduced personal accomplishment. The procedural models of burnout show that burnout and its dimensions have the potential to trigger other psychological processes.

### Burnout and motivation

As mentioned, there are some conflicting findings in the literature regarding the role of burnout in a research context. We advocate for the procedural/over-time aspect of burnout, which could eventually impede the generation of energetic forces to initiate work-related behaviors and, hence, affect their manifestation, direction, intensity, persistence, and duration.

Also, as discussed above, burnt-out workers often experience emotional exhaustion, a detachment from work, adverse reactions to the job, feelings of inadequacy and failure, or reduced personal accomplishment (Maslach et al., 2001; Maslach and Leiter, 2008; Demerouti et al., 2021). These negative factors might lead to ‘developing’ less interest in the job, less “want to” feelings toward putting energy to work (i.e., autonomous motivation), and more “have to” feelings to receive desired outcomes (i.e., controlled motivation). Furthermore, drawing upon the Conservation of Resources (COR) theory (Hobfoll, 1989, 2011), prolonged exposure to stressors or burnout may have an adverse impact on the resources individuals have at any given time (for instance – at work). If this is the case, prolonged (untreated) burnout might lead to resource dwindling and exacerbate negative outcomes, such as reduced will to work, apathy, fatigue, and more. Ultimately, burning out can lead to decreased autonomous (e.g., identification- and integration-based) motivation, but since resources are consistently being depleted, this would also negatively affect other types of motivation (e.g., controlled). In sum, based on the above discussion, we suggest the following hypotheses:

*H1*: Burnout negatively associates with autonomous motivation.

*H2*: Burnout negatively associates with controlled motivation.

### Organizational citizenship behaviors

Organizational citizenship behaviors (OCB), as defined by Tziner et al. (2020b), encompasses voluntary and benevolent behaviors undertaken by employees that go beyond their formal job duties and contractual obligations (e.g., assisting colleagues and superiors, aiding newcomers, increasing active involvement within the organization; Podsakoff et al., 2009). OCB can manifest in two different ways: (1) *OCB-I* (Interpersonal dimension), which involves actions aimed at other members of the organization, such as colleagues or managers (e.g., providing assistance to a coworker without expecting anything in return), and (2) *OCB-O* (Organizational dimension), pertains to actions directed towards the organization itself, such as speaking favorably about the organization to external parties, keeping firm property safe and more (for further reading, see Williams and Anderson, 1991; Urbini et al., 2020).

Antecedents to OCB are plenty, from altruistic dispositions (e.g., Emmerik et al., 2005) and conscientiousness (e.g., Bourdage et al., 2012), high work commitment (e.g., Chun et al., 2013), perceived just workplace (Karriker and Williams, 2009), when employees want their organization to succeed (Lemmon and Wayne, 2015), when they are concerned about their supervisors (Lemmon and Wayne, 2015), or even as a part of impression management (Rioux and Penner, 2001). In the next section, we outline the link between work motivation and OCB.

### OCB and motivation

The relationship between motivation and OCB has been explored in the literature (e.g., Finkelstein, 2011; Soyer et al., 2022). In the context of the current paper, this association is replicated. On the one hand, being autonomously motivated alludes to the enjoyment, positive challenge, and interest the worker derives from their work. As such, it seems almost natural that they would like to contribute to it in various ways, one way of which may be through voluntary behaviors aimed towards helping the workplace and/or its members. On the other hand, a controlled motivation-driven employee would still be more inclined to engage in OCBs as this may be a very good way of self-promotion and impression management (whether tangible/intangible rewards are a consideration). As such, we hypothesize:

*H3*: Autonomous motivation positively associates with OCB-I and OCB-O.

*H4*: Controlled motivation positively associates with OCB-I and OCB-O.

### Work misbehaviors

These refer to “employees’ reducing or withdrawing their input to balance the social exchange process (Greenberg and Scott, 1996), feeling negative toward the organization, feeling less motivated, exhibiting distrust, and even retaliating against the organization (Skarlicki and Folger, 1997), which might manifest as harassment, theft, or sabotage (Bennett and Robinson, 2000; Spector et al., 2006) “(Tabak et al., 2021, p. 10; references are in the original article). WMBs are typically comprised of two dimensions (Bennett and Robinson, 2000; Fox et al., 2001; Dalal, 2005; Shkoler et al., 2019; Tabak et al., 2021). The first is *organizationally* directed WMB (WMB-O), which pertains to behaviors aimed at causing harm to the organization itself, its processes, or its resources. This dimension includes actions such as theft, sabotage, or deliberate withdrawal of effort (Bennett and Robinson, 2000; Dalal, 2005), suseptible to stress and burnout effecrts (e.g., Lebron et al., 2018). The second dimension is *interpersonally* directed WMB (WMB-I), which focuses on behaviors directed towards other members of the workplace colleagues or co-workers, often involving actions like bullying or harassment (Bennett and Robinson, 2000; Fox et al., 2001). The literature showcases a multitude of variables that can predict WMBs from individual differences like cognitive abilities and emotional (in)stability (e.g., Berry et al., 2007) to contextual factors like organizational justice perceptions and job (dis)satisfaction (e.g., Shkoler and Tziner, 2017) and other work stressors, such as bullying or abusive leadership (e.g., Mitchell and Ambrose, 2007; Drory et al., 2022).

### WMB and motivation

Like OCB, the relationship between work motivation and WMB is not new in the literature and will be replicated in the current study. Scholars suggest that individuals are more inclined to engage in WMBs as a coping mechanism for work-related stressors (Diefendorff and Mehta, 2007; Penney and Spector, 2008; Shkoler and Tziner, 2017; Shkoler et al., 2021a,b). The logic behind this association lies in motivation itself; an employee who is motivated to work, irrespective of the source (autonomous vs. controlled), would show less propensity to retaliate or exhibit behaviors that are harmful to the organization or its members and vice versa (a demotivated worker would tend to engage in more WMBs) (van den Broeck et al., 2021). It is important to note for transparency, however, that an amotivated individual would ‘tend’ to become generally indifferent, and since controlled motivation relies on amotivation dimension as well, the direction of this specific association is uncertain and will be hypothesized to be negative to follow the theoretical background’s line of thought. As such, we hypothesize:

*H5*: Autonomous motivation negatively associates with WMB-I and WMB-O.

*H6*: Controlled motivation negatively associates with WMB-I and WMB-O.

### Emotional intelligence

Personality traits and individual differences are critical for managers when staffing and assessing personnel (Bragg and Bowling, 2018; Tziner et al., 2020b) as these factors are intimately and intricately linked to our everyday behaviors. The definitional consensus about emotional intelligence (EI) revolves around a few emotion-related abilities: (1) recognizing and monitoring one’s own and other people’s emotions (2) understanding feelings, and subsequently (3) using emotional information to promote and guide thinking and adapting behavior to suit the environment (Boyatzis, 2009; Joseph and Newman, 2010; Furnham and Taylor, 2020; Tziner et al., 2020a). Many studies associated EI with positive outcomes such as well-being, low emotional exhaustion and burnout, perceived control, low levels of stress, satisfying interpersonal relationships, and commitment to their work (Petrides and Furnham, 2006; Tziner et al., 2020b; Parker et al., 2021; Sanchez-Ruiz et al., 2021). Individuals with high EI regulate their emotions and cope with adversities by creating emotional and behavioral balance utilizing self-control and self-regulation (Mayer et al., 2008; Boyatzis, 2009) that help employees to maintain a positive state of mind (Joseph and Newman, 2010; Shkoler and Tziner, 2017; Tziner et al., 2020b). Control over emotions and maintaining a positive state in the face of adversities can buffer the negative effects of work burnout. Based on COR theory, EI is considered a valuable individual resource that may help us cope with other negative aspects of our lives (or, specifically, in the work context). In this paper, however, we answer the call for research by Davis and Nichols (2016, p. 8) who claim that “there is therefore an urgent need to study “EI in action” by modelling moderating and mediating effects to better understand when and how EI is deployed.”

EI, as mentioned in the Introduction section, is a state-like disposition, meaning it is a trait that can be trained and/or enhanced (e.g., Hodzic et al., 2018; Mattingly and Kraiger, 2019) and is an important factor in profiling employees to better fit them to their job roles (e.g., Tziner et al., 2020a). EI has thus unique practical implications at the workplace, especially for policymakers and HR managers. For instance, training sessions to increase EI for employees in specific relevant industries or occupations (e.g., healthcare) may be of special interest for many workplaces, particularly in the context of interventions for stress reduction and improvement of general mental health (e.g., Hodzic et al., 2018). The malleability of EI is what makes it a great tool for managers and organizations to keep employees at optimal functioning working levels.

### The buffering effect of EI

In the current study, we suggest that EI can moderate the effect of burnout on motivation at work. In line with literature finding beneficial effects of EI, we suggest that high levels of EI decrease the negative effect of burnout on autonomous motivation and its positive effect on controlled motivation. As discussed earlier, high EI individuals can better cope with negative feelings and experiences to maintain a positive state of mind, making them less likely to lose their interest and connection with work itself when they experience burnout. High EI individuals exhibit more awareness and control over their own emotions, and they can utilize this ability to regulate negativity and maintain a more positive mindset. As such, when facing a stressful event, people high in EI would be able to understand the situation better, frame it more positively, and, as a result, cope with it better.

*H7*: EI moderates the negative effect of burnout on autonomous motivation, such that as EI increases, the negative association decreases.

*H8*: EI moderates the negative effect of burnout on controlled motivation, such that as EI increases, the negative association decreases.

### Burnout, motivation, OCB and WMB

As previously discussed, job burnout can deplete the resources of the employee over time, and thus create a motivation deficiency. This decrease in autonomous or controlled motivation might affect the worker’s behavior and might result in increased WMBs and decreased OCBs. In this sense, motivation acts as a mediational mechanism by which work burnout can indirectly impact the expression and engagement in these behaviors at the workplace. Hence, we hypothesize:

*H9*: Autonomous motivation mediates the relationship between Burnout and OCB (I/O) and WMB (I/O).

*H10*: Controlled motivation mediates the relationship between Burnout and OCB (I/O) and WMB (I/O).

The hypotheses and the research model are portrayed in Figure 1.

### Managerial levels

We contend, however, that the relationships described previously would play out differently depending on the managerial level on the employee. Evidently, it is to be expected that each managerial level (e.g., first-level, mid-level, and top-level) would feature different degrees of job resources, demands, motivations, work stressors, and in-role and extra-role behaviors (e.g., Aronson et al., 2005; Kinnunen et al., 2008; Frame et al., 2010; Lundqvist et al., 2013; Giamos et al., 2023). Specifically, managers are expected by others in the organization to invest more time and effort in their work due to the increase in job demands related to their role in the organization. These different levels of investment at work are “built-in” in the role of the manager (Rabenu et al., 2019). Indeed, managers tend to invest more time (e.g., Kinnunen et al., 2008; Shkoler et al., 2017) and effort (e.g., Tziner et al., 2019) in their jobs compared to non-managerial employees. Managers are even organizationally expected to be role models for their subordinates (Rabenu et al., 2019). Consequently, due to having higher work demands, managers are more prone to experience increased exhaustion at work (Blom et al., 2016). Therefore, since the predictor in the model (i.e., burnout; see Figure 1) is highly likely to change across managerial positions, it is fairly plausible that the outcomes would differ as well.

Furthermore, the manager’s job is usually more complex than that of non-managers (e.g., Stam et al., 2010) and the weight personal performance has for managerial roles is higher than for non-managerial ones (Petrou et al., 2017). They are a crucial part of setting the climate of the organization (Rosenhan et al., 1976), and even in determining wage and promotional policies (Viswesvaran et al., 1998). Managers also have, as opposed to non-managers, a more ethical approach in the workplace (Siu and Lam, 2009) and a higher sense of social responsibility at work (Factor et al., 2013). Thus, the literature suggests that managers’ engagement in work behaviors differs across hierarchical levels. To illustrate, *managers* are usually expected to have above-the-norm attendance at work, and so, such behavior will *not* be perceived as extra-role (i.e., OCB) by managers, but will be by common workers. As another example, whereas sharing knowledge and information with coworkers is considered an OCB by *non-managerial* employees, it is regarded as a responsibility in the managerial spheres, as managers are expected to share important details with the chain of command in the organization.

Given these clear differences in attitudes and behaviors between managers and non-managers (see also Samson et al., 2021), ultimately, this will lead each group to have different experiences, demands, and resources at work. As such, in the present study, we expect that the associations in our model will differ depending on the managerial level of participants. Hence, we hypothesize:

*H11*: The relationships specified in Figure 1 will differ across managerial levels (i.e., first-level, mid-level, and top management level).

## Method

### Participants

The sample consists of 840 Romanian managers, in total, separated into three groups based on their managerial level: (1) office/team managers or first-level managers (*n* = 513); (2) heads of departments or mid-level managers (*n* = 220); and (3) top managers and executives (*n* = 107). The research survey was distributed to the general working population in Romania; no specific industry or organization was targeted.

Chi-square tests and one-way ANOVAs assessed *in-situ* demographical differences amongst the managerial groups. The results are presented in Table 1 and indicate that there are no statistically significant differences in the distribution of gender or education level, alluding to the relatively equal representation of both men and women and education in the three managerial levels. Although the groups are of unequal size, they are representative of the managerial population (e.g., there are far fewer executive managers than junior ones in most organizations).

**Table 1:** Descriptive statistics for demographics and managerial group comparison.

	Managerial group	First-level^1^		Mid-level^2^		Top-level^3^		
Demographic	Category	*f*	%	*f*	%	*f*	%	Diff. test
Sex	Woman	262	51.1	109	49.5	47	43.9	χ^2^ (2) = 1.81
	Man	251	48.9	111	50.5	60	56.1	*r_c_* = 0.05
Education	Full high-school	104	20.3	32	14.5	14	13.1	χ^2^ (8) = 14.40,
	Post-secondary	43	8.4	13	5.9	6	5.6	*r_c_* = 0.13
	Bachelor’s degree	211	41.1	88	40.0	45	42.1	
	Master’s degree	154	30.0	86	39.1	40	37.4	
	Doctorate degree	1.0	0.2	1	0.5	2	1.9	
Age _*M* (SD)_	–	31.69 (10.42)		34.90 (11.71)		34.50 (12.54)		*F* = 8.36^***^
Tenure _*M* (SD)_	–	8.72 (8.40)		11.10 (9.73)		12.99 (11.02)		*F* = 12.26^***^

***p < 0.001. Managerial groups: (1) *n* = 513. (2) *n* = 220. (3) *n* = 107. f, frequency (counted). %, relative frequency (percentage). Diff. test, statistical difference test (*F*-test for an ANOVA, chi-square for a Chi-Square test).

### Measures

#### Burnout

We used the Maslach Burnout Inventory (MBI; Maslach and Jackson, 1981), containing 22 items with responses ranging from 1 (*a few times a year*) to 6 (*every day*) (e.g., “I feel emotionally drained from my work”).

#### Emotional intelligence

We used the Trait Emotional Intelligence Questionnaire—Short Form (TEIQue-SF; Petrides, 2009), comprising 30 items with responses ranging from 1 (*very little*) to 6 (*very much*) (e.g., “Expressing my emotions with words is not a problem for me”). Half of the items are reverse-coded. This scale has been found to be reliable and valid in previous studies (see Andrei et al., 2016).

#### Work motivation

We used the Work Extrinsic and Intrinsic Motivation Scale (WEIMS; Tremblay et al., 2009), including 18 items whose responses ranged from 1 (*does not correspond at all*) and 6 (*corresponds exactly*). The *Autonomous* motivation scale consists of 7 items (e.g., “for the satisfaction I experience from taking on interesting challenges”), while the *Controlled* motivation scale comprises 11 items (e.g., “for the income it provides”).

#### Organizational citizenship behaviors

We used the OCB scale from Williams and Anderson (1991), which includes 14 items that are rated on a scale ranging from 1 (*never*) to 6 (*always*). The *Interpersonal* and the *Organizational* dimensions both contain 7 items (e.g., “conserves and protects organizational property;” OCB-O).

#### Work misbehaviors

We used the Interpersonal and Organizational Deviance Scale (IODS; Bennett and Robinson, 2000), which has 19 items rated on a scale ranging from 1 (*never*) to 6 (*every day*). The *Interpersonal* dimension consists of 7 items (e.g., “said something hurtful to someone at work”), while the *Organizational* dimension comprises 11 items (e.g., “taken property from work without permission”). This scale has been found to be reliable and valid in previous studies (see Berry et al., 2007).

Table 2 presents descriptive statistics (means and standard deviations) of the variables per managerial group. One-way ANOVAs were conducted to test the statistical differences among the groups. As can be seen, the groups are unbiased as there are no *a-priori* differences between them, supporting the sample’s representativeness.

**Table 2:** Descriptive statistics for the managerial groups.

	First-level^2^			Mid-level^3^			Top-level^4^			
Variable	α	*M*	*SD*	α	*M*	*SD*	α	*M*	*SD*	Diff. test
Burnout	0.89	2.72	0.75	0.89	2.75	0.76	0.88	2.63	0.75	0.99
Emotional intelligence	0.91	4.19	0.92	0.93	4.23	0.98	0.93	4.27	1.01	0.39
Autonomous motivation	0.91	4.33	1.02	0.91	4.35	1.00	0.93	4.41	1.04	0.29
Controlled motivation	0.76	3.91	0.79	0.80	3.96	0.86	0.76	3.86	0.80	0.60
OCB-I	0.84	4.08	0.97	0.85	4.28	0.96	0.87	4.10	0.99	2.91
OCB-O	0.60	3.47	0.79	0.65	3.66	0.83	0.65	3.52	0.85	3.08
WMB-I	0.85	2.42	1.04	0.88	2.53	1.15	0.86	2.53	1.12	1.03
WMB-O	0.95	2.14	1.16	0.97	2.25	1.28	0.96	2.21	1.25	0.69

(1) *n* = 513. (2) *n* = 220. (3) *n* = 107. α, Cronbach’s alpha reliability coefficient; OCB, organizational citizenship behaviors; WMB, work misbehaviors. Diff. test, one-way ANOVA *F*-test.

#### Control variables

We used controlled variables as recommended by Bernerth and Aguinis (2016) as well as Martins et al. (2010). The analyses controlled for the effects of three demographics generally associated with work and managerial positions: gender (0 = man; 1 = woman), education level (1 = high school; 2 = post-secondary; 3 = Bachelor’s degree; 4 = Master’s degree; 5 = Doctorate degree), and tenure (in years).

### Procedure

The pencil-and-paper research survey was distributed to working individuals through informal networks. The convenience sample targeted no specific industry or organization. After data collection, SPSS (v. 28) and AMOS (v. 28) statistical packages and PROCESS (v. 3.5.2) macro were used to examine the data.

### Data screening and analyses

Outlier exploration using Mahalanobis and Cook’s distances revealed a minimal number of outliers (*n* = 8). After testing the research model and hypotheses with and without the outliers, there was no noticeable difference between the two analyses. As such, we decided to retain the outliers, trying to keep to the original raw data and promote higher statistical power. The group sizes mentioned in the Participants subsection represents the final sample.

The data analyses consist of zero-order correlations computed in SPSS to gauge the bivariate baseline relationships amongst the variables. AMOS software was utilized to assess the overall structural model fit. Finally, PROCESS model #7 is utilized to examine the moderated mediation relations depicted in Figure 1 due to its superior algorithms in conducting and visualizing moderation analyses, using heteroscedasticity-consistent standard errors (SE) (e.g., Hayes and Cai, 2007; Shkoler and Kimura, 2020), to ensure that the parameter estimates’ covariance matrix will not be biased and/or inconsistent given deviations from heteroscedasticity. The analyses were calculated with bootstrapping based on 5,000 re-samples of the data and a 95% bias-corrected confidence interval (CI) and controlling for the effects of gender, education level, and tenure.

## Results

As a first step, zero-order (Pearson) correlations were calculated to examine the bivariate associations amongst the variables. The results are shown on Table 3. Multivariate analysis of the research model shows that *Burnout* negatively associates with *Autonomous Motivation* at all managerial levels, and negatively associates with *Controlled Motivation* only among first-level managers. As the bivariate results are not the core of the current study, the full group-level *correlational analysis* is presented in Appendix A, to facilitate readability.

**Table 3:** Pearson correlation matrix for first-level managers (outside parenthesis; *n* = 513), mid-level (in parenthesis; *n* = 220) and top-level [in brackets; *n* = 107].

	**1**	**2**	**3**	**4**	**5**	**6**	**7**
Burnout	–						
EI	−0.69^***^ (−0.68^***^) [−0.64^***^]	–					
*Aut*. Mot.	−0.41^***^ (−0.26^***^) [−0.46^***^]	0.22^***^ (0.11) [0.14]	–				
*Con.* Mot.	−0.11^**^ (−0.04) [−0.10]	−0.08^***^ (−0.09) [−0.27^**^]	0.68^***^ (0.73^***^) [0.69^***^]	–			
OCB-I	−0.20^***^ (−0.06) [−0.26^**^]	0.10 (−0.04) [0.08]	0.39^***^ (0.50^***^) [0.31^**^]	0.31^***^ (0.41^***^) [0.26^**^]	–		
OCB-O	0.12^**^ (0.19^**^) [0.07]	−0.16^***^ (−0.33^***^) [−0.19^*^]	0.08 (0.29^***^) [0.16]	0.16^***^ (0.40^***^) [0.35^***^]	0.46^***^ (0.52^***^) [0.48^***^]	–	
WMB-I	0.51^***^ (0.51^***^) [0.46^***^]	−0.55^***^ (−0.56^***^) [−0.54^***^]	−0.26^***^ (−0.14^*^) [−0.12]	−0.07 (0.08) [0.25^*^]	−0.09^*^ (0.01) [0.15]	0.21^***^ (0.29^***^) [0.41^***^]	–
WMB-O	0.57^***^ (0.53^***^) [0.53^***^]	−0.56^***^ (−0.56^***^) [−0.70^***^]	−0.34^***^ (−0.20^**^) [−0.26^**^]	−0.09^*^ (0.06) [0.13]	−0.17^***^ (−0.08) [−0.07]	0.24^***^ (0.31^***^) [0.32^**^]	0.85^***^ (0.88^***^) [0.80^***^]

*p < 0.05, **p < 0.001, ***p < 0.001. Aut. Mot., autonomous motivation; Con. Mot., controlled motivation; OCB, organizational citizenship behaviors; WMB, work misbehaviors; I, interpersonal dimension; O, organizational dimension.

### Moderated-mediation analysis

**Figure 2:**
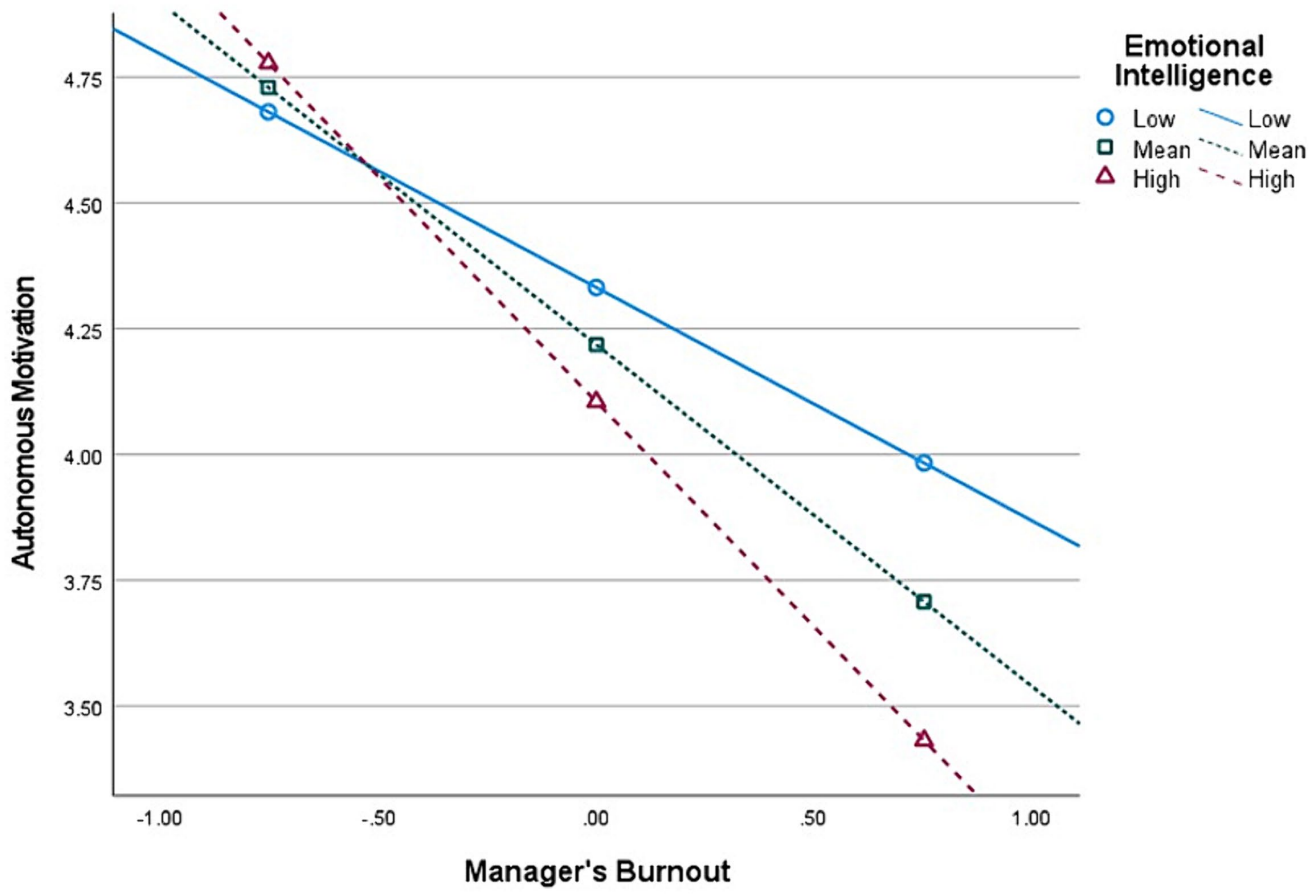
Interaction (Burnout × Emotional Intelligence) graph in predicting Autonomous Motivation, for *first-level* managers’ group (*n* = 513).

**Figure 3:**
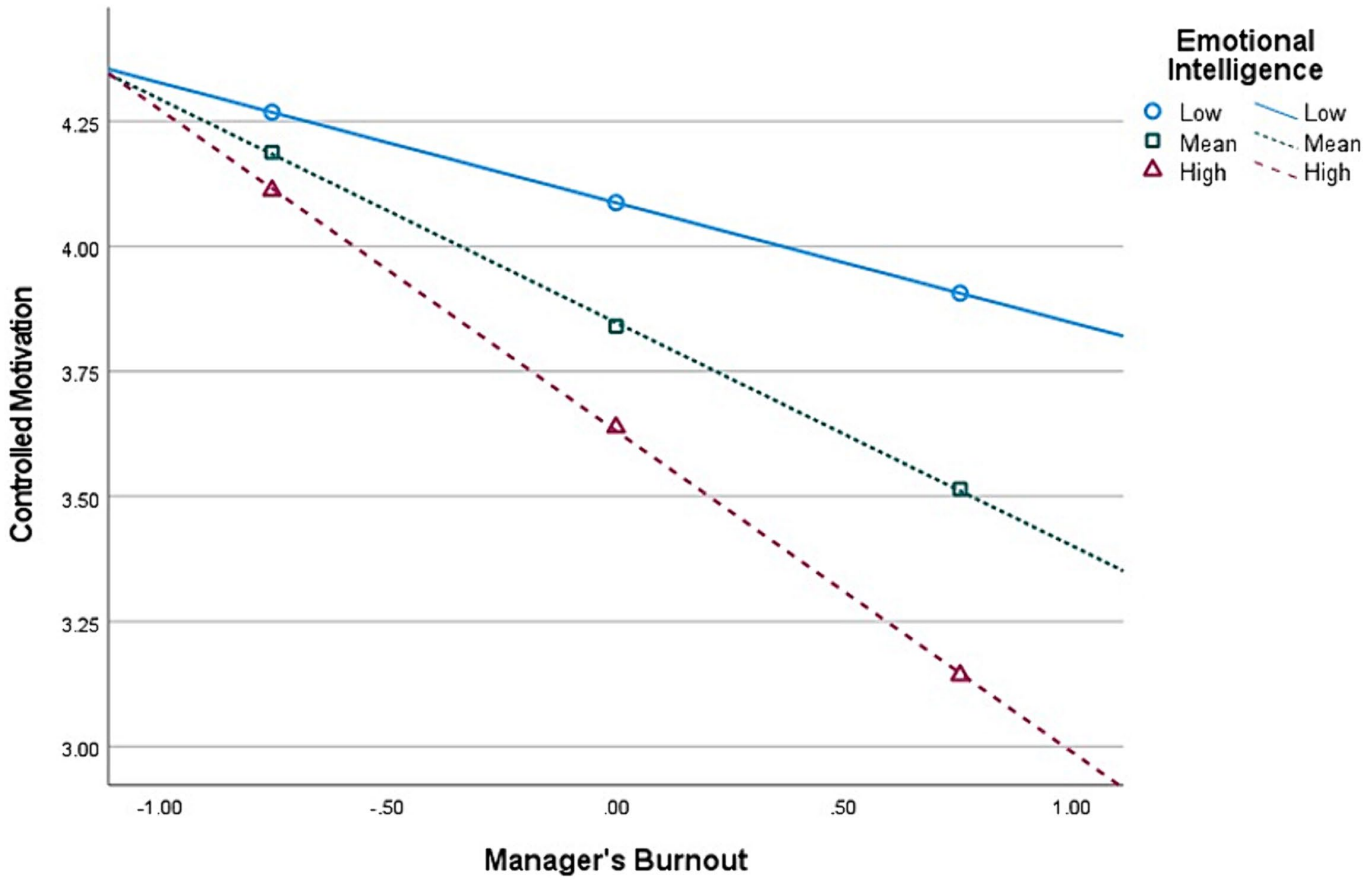
Interaction (Burnout × Emotional Intelligence) graph in predicting Controlled Motivation, for *first-level* managers’ group (*n* = 513).

**Figure 4:**
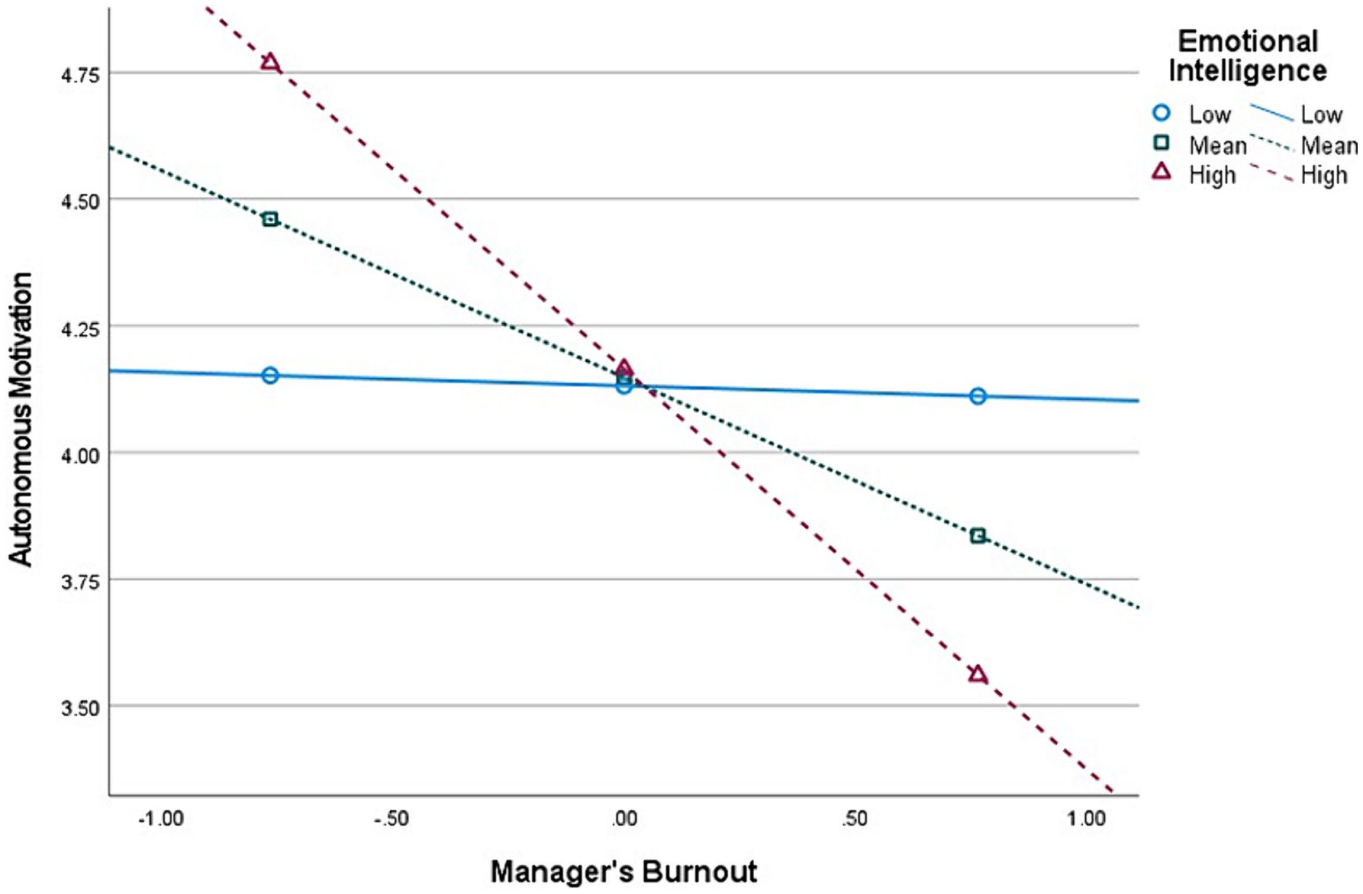
Interaction (Burnout × Emotional Intelligence) graph in predicting Autonomous Motivation, for *mid-level* managers’ group (*n* = 220).

**Figure 5:**
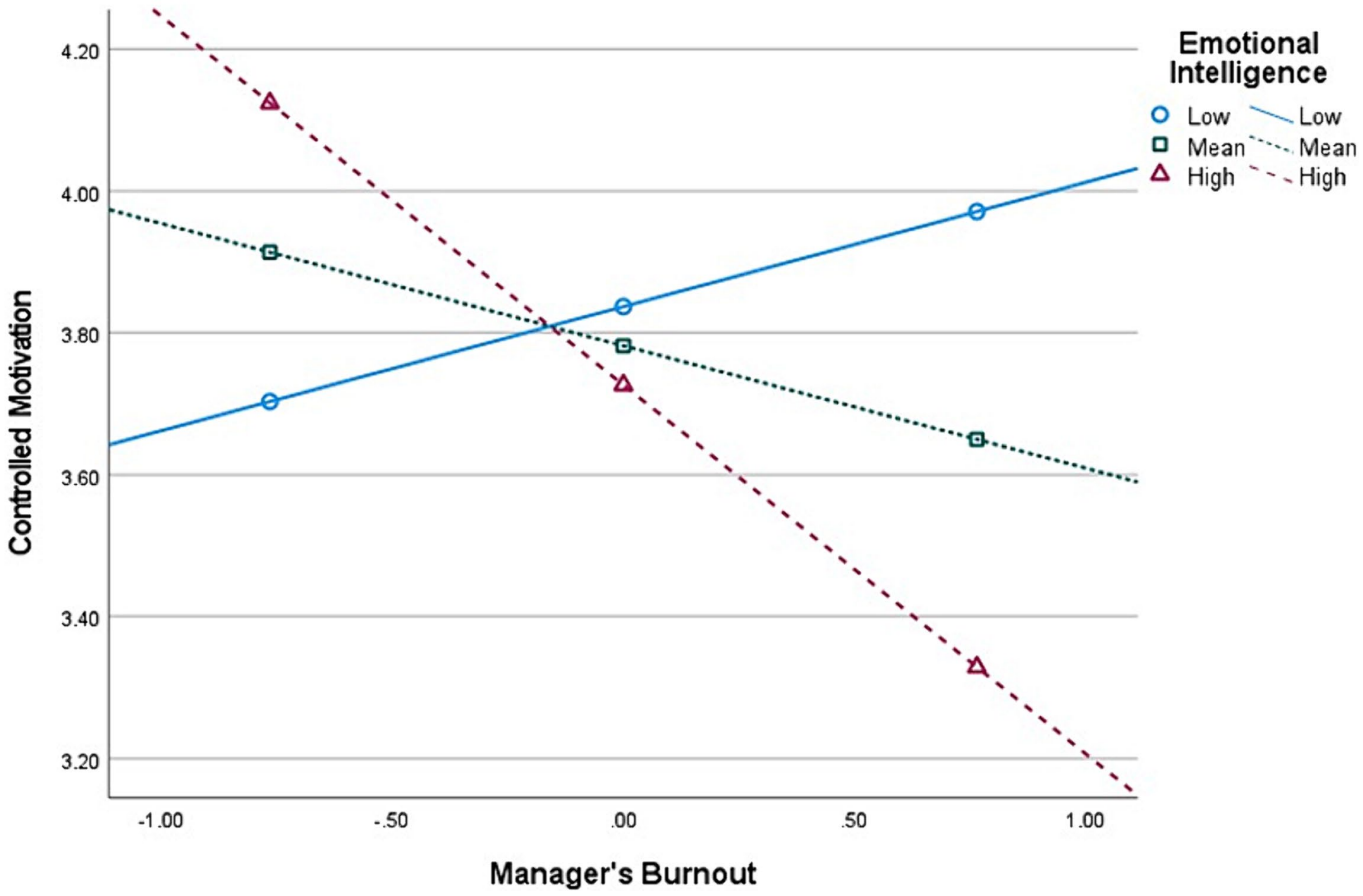
Interaction (Burnout × Emotional Intelligence) graph in predicting Controlled Motivation, for *mid-level* managers’ group (*n* = 220).

**Figure 6:**
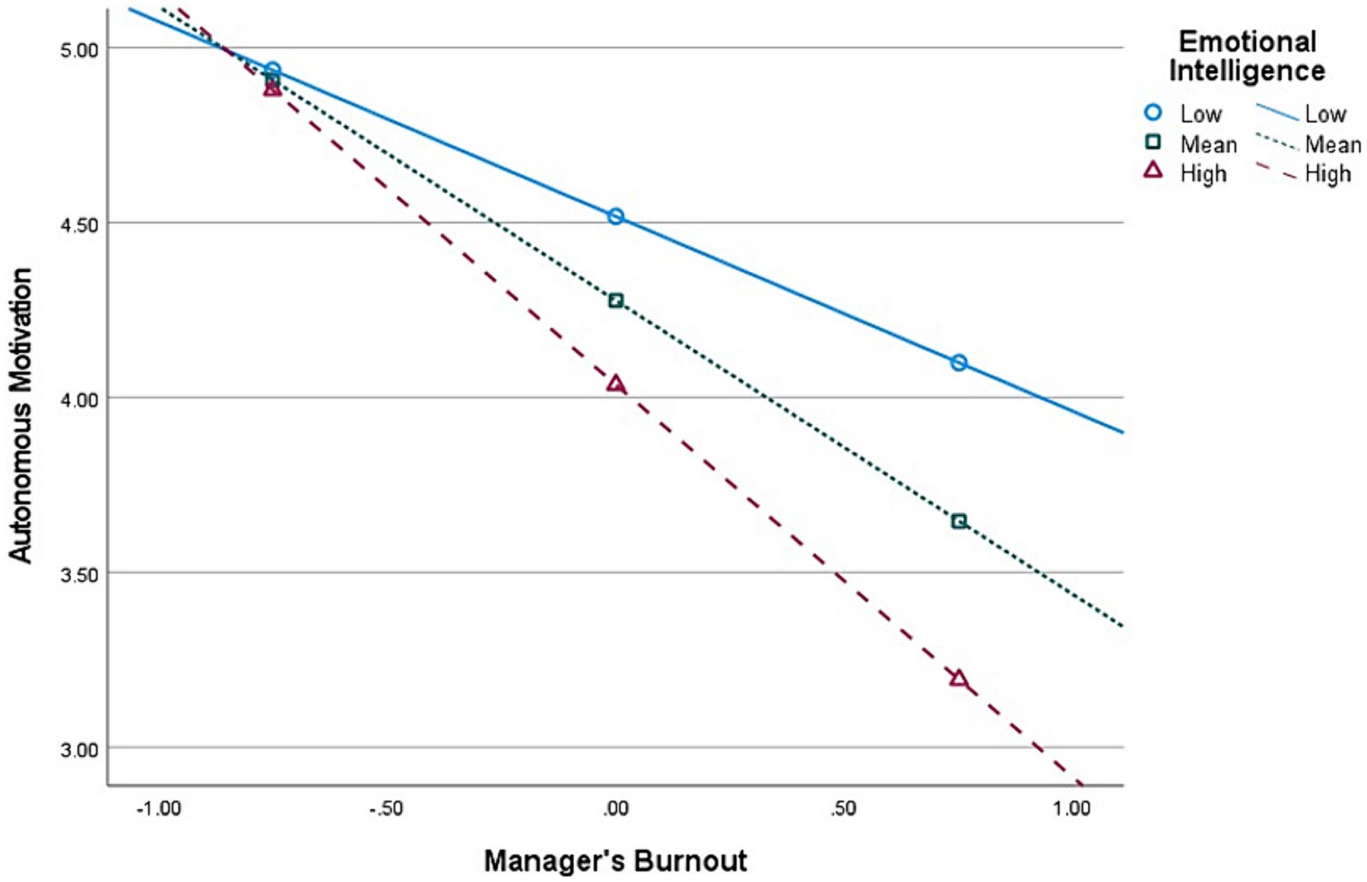
Interaction (Burnout × Emotional Intelligence) graph in predicting Autonomous Motivation, for *top-level* managers’ group (*n* = 107).

**Figure 7:**
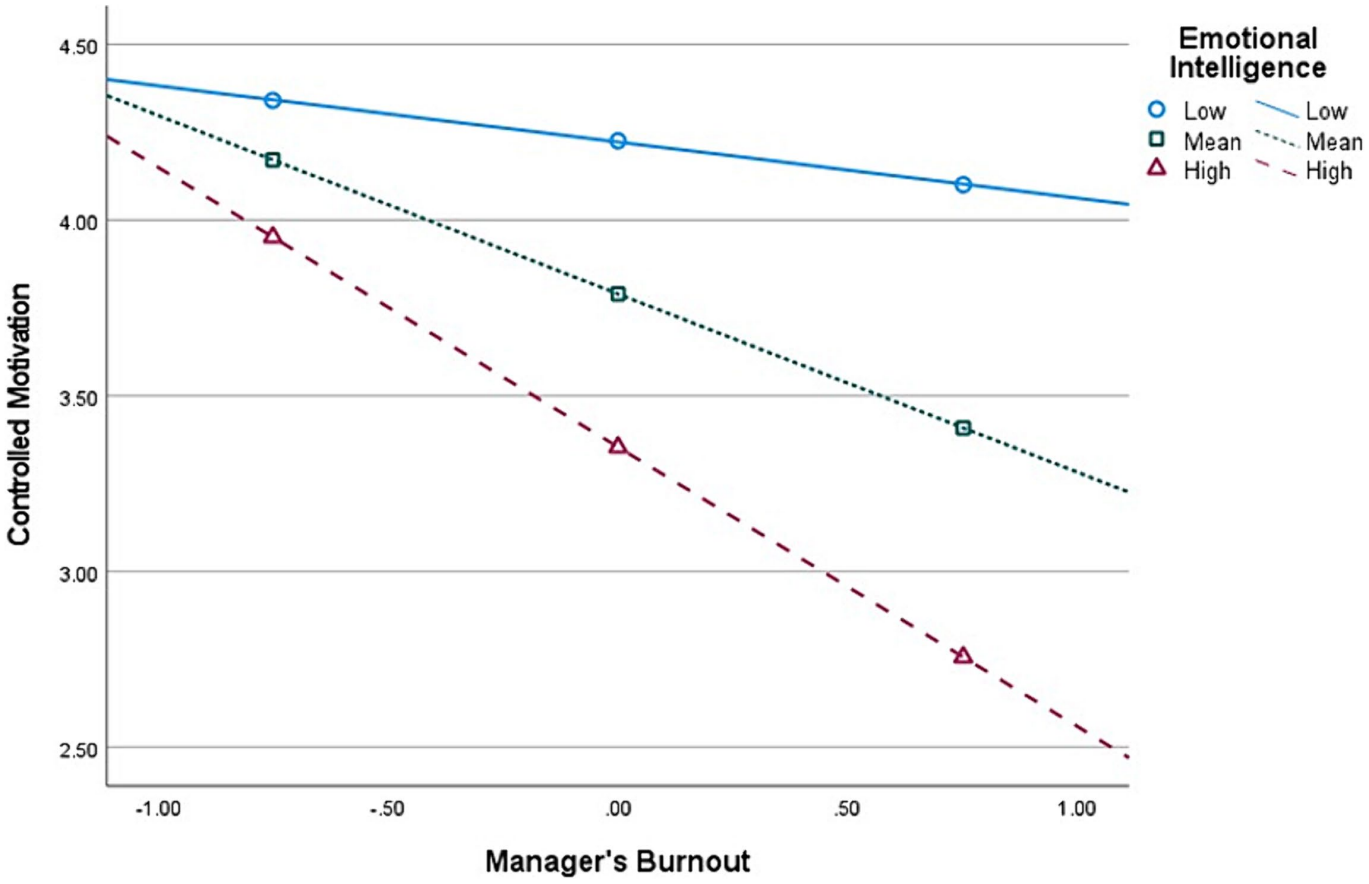
Interaction (Burnout × Emotional Intelligence) graph in predicting Controlled Motivation, for *top-level* managers’ group (*n* = 107).

Results from a multiple-group structural equation modelling analysis revealed that the most constrained (i.e., the more structurally parsimonious model) model is preferred to the unconstrained model, with an excellent fit (Byrne, 2010): χ^2^ (60) = 165.722, *p* = 0.000, CFI = 0.961, TLI = 0.959, SRMR = 0.054, RMSEA _[90% CI]_ = 0.046 [0.038–0.054], *p* = 0.783. The results from the moderated-mediation analyses in PROCESS are shown in Tables 4–6. The moderating effects are depicted in Figures 2–7.

**Table 4:** Moderated-mediation results for predicting OCB and WMB.

Managerial level	First-level^1^		Mid-level^2^		Top-level^3^	
Path	*b*	*SE*	*b*	*SE*	*b*	*SE*
Work burnout → Autonomous motivation	−0.68^***^	0.08	−0.41^***^	0.09	−0.84^***^	0.15
Emotional intelligence → Autonomous motivation	−0.12	0.07	0.02	0.08	−0.24	0.13
*INT*_(E.I. × Burnout)_ → Autonomous motivation	−0.23^***^	0.05	−0.39^***^	0.06	−0.28^**^	0.09
Work burnout → Controlled motivation	−0.37^***^	0.07	−0.17	0.10	−0.51^***^	0.07
Emotional intelligence → Controlled motivation	−0.27^***^	0.06	−0.06	0.09	−0.43^***^	0.06
*INT*_(E.I. × Burnout)_ → Controlled motivation	−0.14^***^	0.06	−0.35^***^	0.06	−0.15^*^	0.06
**Work burnout**_**(total)**_ →**OCB-I**	**−0.26** ^***^	**0.06**	**−0.08**	**0.09**	**−0.34** ^**^	**0.12**
Work burnout _(direct)_ → OCB-I	−0.10	0.06	−0.10	0.07	−0.25	0.16
Autonomous motivation → OCB-I	0.27^***^	0.06	0.45^***^	0.08	0.11	0.14
Controlled motivation → OCB-I	0.14	0.07	0.06	0.10	0.21	0.16
**Work burnout**_**(total)**_ →**OCB-O**	**0.13** ^**^	**0.05**	**0.21** ^**^	**0.07**	**0.08**	**0.11**
Work burnout _(direct)_ → OCB-O	0.15^**^	0.05	0.26^***^	0.07	0.08	0.15
Autonomous motivation → OCB-O	0.02	0.05	0.11	0.07	−0.11	0.12
Controlled motivation → OCB-O	0.16^**^	0.05	0.29^**^	0.09	0.48^**^	0.15
**Work burnout**_**(total)**_ →**WMB-I**	**0.70** ^***^	**0.05**	**0.77** ^***^	**0.09**	**0.69** ^***^	**0.13**
Work burnout _(direct)_ → WMB-I	0.66^***^	0.07	0.72^***^	0.10	0.55^***^	0.15
Autonomous motivation → WMB-I	−0.07	0.06	−0.19	0.11	−0.24	0.15
Controlled motivation → WMB-I	0.06	0.07	0.28^*^	0.12	0.60^***^	0.18
**Work burnout**_**(total)**_ →**WMB-O**	**0.87** ^***^	**0.06**	**0.89** ^***^	**0.10**	**0.89** ^***^	**0.14**
Work burnout _(direct)_ → WMB-O	0.77^***^	0.07	0.80^***^	0.11	0.66^***^	0.16
Autonomous motivation → WMB-O	−0.21^***^	0.06	−0.35^**^	0.11	−0.42^**^	0.16
Controlled motivation → WMB-O	0.15^*^	0.07	0.40^**^	0.13	0.63^***^	0.17

*p < 0.05, **p < 0.001, ***p < 0.001. (1) *n* = 513. (2) *n* = 220. (3) *n* = 107. *b*, unstandardized regression coefficient; SE, standard error; OCB, organizational citizenship behaviors; OCB-I, interpersonal dimension of OCB; OCB-O, organizational dimension of OCB; WMB, work misbehaviors; WMB-I, interpersonal dimension of WMB; WMB-O, organizational dimension of WMB; INT, interaction effect; E.I., emotional intelligence; Aut. Mot., autonomous motivation; Cont. Mot., controlled motivation. As depicted in Figure 1, Burnout = predictor; Autonomous/Controlled Motivations = mediators; Emotional Intelligence = moderator; OCB (I/O) + WMB (I/O) = outcomes.

**Table 5:** Conditional indirect effects analyses for predicting OCB.

Managerial level	First-level^1^		Mid-level^2^		Top-level^3^	
Conditional indirect path	Effect	[LL, UL]	Effect	[LL, UL]	Effect	[LL, UL]
***Low* emotional intelligence**						
Work burnout → *Autonomous* motivation → OCB-I	**−0.12**	**−0.21, −0.06**	−0.01	−0.11, 0.08	−0.06	−0.27, 0.11
***Mean* emotional intelligence**						
Work burnout → *Autonomous* motivation → OCB-I	**−0.18**	**−0.28, −0.10**	**−0.18**	**−0.31, −0.08**	−0.10	−0.35, 0.15
***High* emotional intelligence**						
Work burnout → *Autonomous* motivation → OCB-I	**−0.24**	**−0.37, −0.13**	**−0.36**	**−0.55, −0.19**	−0.13	−0.47, 0.18
***Low* emotional intelligence**						
Work burnout → *Controlled* motivation → OCB-I	−0.03	−0.09, 0.002	0.01	−0.03, 0.06	−0.07	−0.22, 0.05
***Mean* emotional intelligence**						
Work burnout → *Controlled* motivation → OCB-I	−0.05	−0.11, 0.003	−0.01	−0.07, 0.03	−0.11	−0.28, 0.06
***High* emotional intelligence**						
Work burnout → *Controlled* motivation → OCB-I	−0.07	−0.15, 0.005	−0.03	−0.15, 0.07	−0.14	−0.38, 0.07
***Low* emotional intelligence**						
Work burnout → *Autonomous* motivation → OCB-O	−0.01	−0.06, 0.04	−0.01	−0.03, 0.02	0.06	−0.05, 0.35
***Mean* emotional intelligence**						
Work burnout → *Autonomous* motivation → OCB-O	−0.02	−0.08, 0.05	−0.04	−0.11, 0.01	0.09	−0.10, 0.38
***High* emotional intelligence**						
Work burnout → *Autonomous* motivation → OCB-O	−0.02	−0.11, 0.07	−0.09	−0.21, 0.02	0.12	−0.14, 0.44
***Low* emotional intelligence**						
Work burnout →*Controlled* motivation → OCB-O	**−0.04**	**−0.09, −0.01**	0.05	−0.02, 0.12	**−0.17**	**−0.30, −0.04**
***Mean* emotional intelligence**						
Work burnout → *Controlled* motivation → OCB-O	**−0.06**	**−0.11, −0.02**	−0.05	−0.14, 0.01	**−0.24**	**−0.30, −0.07**
***High* emotional intelligence**						
Work burnout → *Controlled* motivation → OCB-O	**−0.08**	**−0.15, −0.02**	**−0.15**	**−0.30, −0.04**	**−0.32**	**−0.30, −0.08**

(1) *n* = 513. (2) *n* = 220. (3) *n* = 107; OCB, organizational citizenship behaviors; OCB-I, interpersonal dimension of OCB; OCB-O, organizational dimension of OCB; LL and UL, lower limit and upper limit, respectively, of a 95% bias-corrected confidence interval (5,000 re-samples). To facilitate readability, bolded results are statistically significant.

**Table 6:** Conditional indirect effects analyses for predicting WMB.

Managerial level	First-level^1^		Mid-level^2^		Top-level^3^	
Conditional indirect path	Effect	[LL, UL]	Effect	[LL, UL]	Effect	[LL, UL]
***Low* emotional intelligence**						
Work burnout → *Autonomous* motivation →WMB-I	0.03	−0.02, 0.08	0.01	−0.04, 0.06	0.13	−0.02, 0.37
***Mean* emotional intelligence**						
Work burnout → *Autonomous* motivation → WMB-I	0.05	−0.03, 0.12	0.08	−0.01, 0.18	0.20	−0.04, 0.45
***High* emotional intelligence**						
Work burnout → *Autonomous* motivation → WMB-I	0.06	−0.04, 0.15	0.15	−0.02, 0.33	0.27	−0.04, 0.59
***Low* emotional intelligence**						
Work burnout → *Controlled* motivation → WMB-I	−0.01	−0.05, 0.02	0.05	−0.02, 0.13	**−0.21**	**−0.48, −0.05**
***Mean* emotional intelligence**						
Work burnout → *Controlled* motivation → WMB-I	−0.02	−0.08, 0.02	−0.05	−0.15, 0.01	**−0.31**	**−0.57, −0.10**
***High* emotional intelligence**						
Work burnout → *Controlled* motivation → WMB-I	−0.03	−0.11, 0.03	**−0.15**	**−0.32, −0.02**	**−0.40**	**−0.76, −0.11**
***Low* emotional intelligence**						
Work burnout → *Autonomous* motivation → WMB-O	**0.10**	**0.04, 0.17**	0.01	−0.07, 0.10	**0.24**	**0.05, 0.53**
***Mean* emotional intelligence**						
Work burnout → *Autonomous* motivation → WMB-O	**0.14**	**0.06, 0.22**	**0.14**	**0.05, 0.26**	**0.35**	**0.11, 0.63**
***High* emotional intelligence**						
Work burnout → *Autonomous* motivation → WMB-O	**0.18**	**0.08, 0.29**	**0.28**	**0.11, 0.46**	**0.47**	**0.13, 0.82**
***Low* emotional intelligence**						
Work burnout → *Controlled* motivation → WMB-O	**−0.04**	**−0.09, −0.01**	0.07	−0.03, 0.18	**−0.22**	**−0.51, −0.05**
***Mean* emotional intelligence**						
Work burnout → *Controlled* motivation → WMB-O	**−0.05**	**−0.12, −0.01**	−0.07	−0.19, 0.01	**−0.32**	**−0.59, −0.11**
***High* emotional intelligence**						
Work burnout → *Controlled* motivation → WMB-O	**−0.07**	**−0.16, −0.01**	**−0.21**	**−0.40, −0.05**	**−0.41**	**−0.74, −0.12**

(1) *n* = 513. (2) *n* = 220. (3) *n* = 107. WMB, work misbehaviors; WMB-I, interpersonal dimension of WMB; WMB-I, interpersonal dimension of WMB; WMB-O, organizational dimension of WMB; LL and UL, lower limit and upper limit, respectively, of a 95% bias-corrected confidence interval (5,000 re-samples). To facilitate readability, bolded results are statistically significant.

Table 4 reports several statistical findings. First, through optimal functioning, we can see that among *first-level* managers, Burnout associates negatively with OCB-I but positively with OCB-O, and positively relates to both WMB-I and WMB-O. Among *mid-level* managers, Burnout has no direct link to OCB-I but positively associates with OCB-O, WMB-I and WMB-O. At the executive level (top-level), Burnout associates negatively with OCB-I but not with OCB-O, and positively relates to both WMB-I and WMB-O.

Second, E.I. moderates the relationship between Burnout and *Autonomous* Motivation, at all managerial levels. The graphical representations of these significant interactions are depicted in Figures 2, 4, 6. Additionally, E.I. moderates the relationship between Burnout and *Controlled* Motivation, at all managerial levels. The graphical representations of these interactions are depicted in Figures 3, 5, 7. Of note, slopes were generated on three E.I. levels: (1) Low (1 SD above the mean) (2) Mean (0 SD) and (3) High (1 SD above the mean).

*Contrary to our initial hypotheses* (H7–H8), Figure 2 shows that, for *first-level* managers, as E.I. increases, the negative association between Burnout and Autonomous Motivation increases in strength as well. Further, Figure 3 shows that as E.I. increases, the negative association between Burnout and Controlled Motivation also increases in strength.

*Contrary to our initial hypotheses* (H7–H8), Figure 4 shows that, for *mid-level* managers, as E.I. increases, the association between Burnout and Autonomous Motivation becomes more negative (notably, at low E.I. level, this relationship is non-significant). Moreover, Figure 5 shows that as E.I. increases, the association between Burnout and Controlled Motivation becomes considerably more negative (notably, at low E.I. level, this relationship is *positive*).

*Contrary to our initial hypotheses* (H7–H8), Figure 5 shows that, for *top-level* managers, as E.I. increases, the negative association between Burnout and Autonomous Motivation increases in strength as well. In addition, Figure 6 shows that as E.I. increases, the negative association between Burnout and Controlled Motivation also increases in strength.

To further examine moderated mediation relations, conditional indirect effects estimation, using bootstrapping, and associated CIs were calculated. The results are presented in Tables 5, 6 for OCB and WMB, respectively. The full list of results from Tables 5, 6 are depicted in Appendix B, to facilitate readability. Results indicate that only several indirect effects were statistically significant. However, as a “rule” in the findings, at higher levels of E.I. the indirect effect itself increases in magnitude (positively or negatively). Table 7 summarizes the results of all the analyses, per hypothesis.

**Table 7:** Summary of the results per hypothesis, per managerial group.

#	Hypothesis/Path	First-level	Mid-level	Top-level
1	Burnout negatively associates with autonomous motivation.	Supported	Supported	Supported
2	Burnout negatively associates with controlled motivation.	Supported	*N.S.*	*N.S.*
3.1	Autonomous motivation positively associates with OCB-I	Supported	*N.S.*	*N.S.*
3.2	Autonomous motivation positively associates with OCB-O	Contrary	Contrary	Contrary
4.1	Controlled motivation positively associates with OCB-I	Supported	*N.S.*	*N.S.*
4.2	Controlled motivation positively associates with OCB-O	*N.S.*	Supported	*N.S.*
5.1	Autonomous motivation positively associates with WMB-I	Supported	Supported	Supported
5.2	Autonomous motivation positively associates with WMB-O	Supported	Supported	Supported
6.1	Controlled motivation positively associates with WMB-I	Supported	Supported	*N.S.*
6.2	Controlled motivation positively associates with WMB-O	Supported	Supported	Supported
7	EI increases, the negative burnout-autonomous motivation association decreases.	Contrary	Contrary	Contrary
8	EI increases, the negative burnout-controlled motivation association decreases.	Contrary	Contrary	Contrary
9.1	Autonomous motivation mediates the relationship between Burnout and OCB (I)	p-Supported	p-Supported	*N.S.*
9.2	Autonomous motivation mediates the relationship between Burnout and OCB (O)	*N.S.*	*N.S.*	*N.S.*
9.3	Autonomous motivation mediates the relationship between Burnout and WMB (I)	*N.S.*	*N.S.*	*N.S.*
9.4	Autonomous motivation mediates the relationship between Burnout and WMB (O)	p-Supported	p-Supported	p-Supported
10.1	Controlled motivation mediates the relationship between Burnout and OCB (I)	*N.S.*	*N.S.*	*N.S.*
10.2	Controlled motivation mediates the relationship between Burnout and OCB (O)	p-Supported	Supported	p-Supported
10.3	Controlled motivation mediates the relationship between Burnout and WMB (I)	*N.S.*	p-Supported	p-Supported
10.4	Controlled motivation mediates the relationship between Burnout and WMB (O)	p-Supported	p-Supported	p-Supported
11	Distinct relationships in H1-H10 at the first, mid, and top managerial levels	Full support		

Supported., statistically significant relationships based on the results emanating from the current study, meaning the hypothesized association is supported. p-Supported, cases where the hypothesized link is only partially supported for any reason. Partially supported. Contrary, cases where the hypothesized relationship was statistically significant but opposite to the direction of association in the hypothesis. N.S., non-significant (non-supported) cases.

## Discussion

The current study was set up to investigate four main goals: (1) explore the predictive capacity of job burnout for work behavior; (2) examine the indirect (mediational) mechanism of autonomous and controlled work motivation; (3) test the buffering effect of emotional intelligence (EI) on adverse experiences; and (4) explore intricate differences amongst three managerial levels (i.e., first-level, mid-level, and top-level). The findings partially support the research model (Figure 1), yet we encountered unexpected results concerning some of our hypotheses. Since the statistical analyses were rather complex, in the following sections, we briefly summarize the relevant findings and discuss their implications. However, it is important to note that the hypothesized differences amongst managerial levels (H11) were fully supported (i.e., there are clear disparities in the associations among our variables across the three managerial positions).

### Bivariate hypotheses (H1–H6)

The findings related to the bivariate hypotheses found partial support based on the managerial level. To reduce complexity, we focus on bivariate hypotheses in line with the notion that burnout, a chronic response to workplace stress, can erode motivation. First, *Hypothesis 1* (i.e., burnout negatively associates with Autonomous motivation) was supported at each managerial level, showing that as burnout increases, the more self-determined forms of motivation decrease across all levels of management. As burnout progresses, individuals may experience a decline in their internal drive to engage in tasks willingly. Second, *Hypothesis 2* (i.e., burnout negatively associates with Controlled motivation) was supported only for the first-level managers. These findings suggest that burnout affects externally regulated or controlled motivation, specifically for those in entry-level management positions. This could be due to the unique stressors, needs, and responsibilities of first-level management roles. For instance, first-level managers have more functional and relational (e.g., subordinates, clients) responsibilities and fewer resources to adjust their work (e.g., shorten their workday) than higher managers (Lundqvist et al., 2013). This finding aligns with the idea that burnout is not only a physical or mental health concern but also has implications for motivational processes and can diminish autonomous and controlled motivation. It is important to note that we observed that burnout is more strongly associated with Autonomous motivation than Controlled motivation, suggesting a more detrimental effect of burnout on autonomous forms of motivation than controlled ones.

### The buffering effect of EI (H7–H8)

Contrary to our hypotheses regarding the moderating effect of EI on the relationship between burnout and autonomous motivation (i.e., H7) and controlled motivation (H8), the results were the opposite. Instead of the buffering effect we expected, EI *increased* the negative relationship between burnout and both Autonomous and Controlled work motivation at each managerial level. Furthermore, an even more unexpected finding, portrayed in Figure 5, is that at the lowest level of EI, the relationship between burnout and Controlled motivation was *positive* and became more negative with increasing degrees of EI. These unique and counterintuitive findings are initially perplexing, yet they align with extant literature on the dark side of emotional intelligence. The construct is evidently and historically framed as positive and as having positive outcomes. Nevertheless, in different contexts or in conjunction with other dispositions, EI might induce adverse outcomes as well (e.g., Davis and Nichols, 2016; Wang and Lei, 2022; Shahzad et al., 2023). Recent studies on EI show that while traditionally, EI is touted as the panacea for a satisfying and successful life (e.g., Tziner et al., 2020a,b), a growing body of research has begun identifying contexts where EI is not only unhelpful but harmful to the person or their organizations and colleagues, suggesting a dark side to the construct (for a review, see Davis and Nichols, 2016). We wish to offer a plausible explanation for these unique results that coincide with Davis and Nichols’s (2016) review. Individuals *high* in EI are attentive to their affective states and environmental emotional cues. We argue that this makes them hyper-aware of their and others’ cognitive-affective states, which could potentially overwhelm them during work, as emotional and social cues may constantly trigger them during working hours (e.g., “hyper-awareness of negative emotional information overwhelms their capacity to regulate. Performance decreases due to regulatory overload in applied contexts”; Davis and Nichols, 2016, p. 8). On the other hand, people with *low* EI tend to exhibit less awareness of both positive and negative aspects of their day-to-day lives. In this instance, it is a paraphrase of “ignorance is bliss,” as they are less likely to be sufficiently aware of negative experiences, such as burnout, stress, unethical happenstance, politics, etc. As such, we posit that these have a meeker impact on individuals with low EI than high EI, as they are less aware of them (e.g., it is as if they do not realize they are burnt-out), thus keeping their psyche and mental capacity unhinged. In other words, in the current paper, EI *augments* the negative relationship between burnout and work motivation. This alludes to the fact that “too much from anything is not good,” such as in the case of EI. Coincidentally, those high in EI but simultaneously low on (emotional) self-confidence might have more adverse experiences than others (see Davis and Nichols, 2016). Other intriguing findings lie in the moderated mediation analysis with EI.

### Moderated-mediation results (H9–H10)

Hypotheses 9 and 10 received partial support depending on the managerial level, EI level, and the behavioral index of functioning (i.e., OCB-I, OCB-O, WMB-I, WMB-O).

#### Burnout and the interpersonal dimension of OCB (OCB-I)

The mediational relationship of burnout through Autonomous Motivation and *OCB-I* (H9.1) was supported for first- and mid-level managers at all levels of EI (except for mid-level managers with low EI). We did not find support for the mediational role of Controlled Motivation in this relationship (H10.1). These findings suggest that burnout, as a well-being indicator of malfunctioning, can lead to reduced voluntary behavior toward colleagues or supervisors through an increase in first- and mid-level managers’ autonomous motivation. So, the mechanism suggests that an upsurge in malfunctioning can adversely impact optimal functioning by affecting autonomous motivation. The absence of support for the mediational role of Controlled Motivation may be due to the unique nature of OCB-I, which might be more closely tied to internal sources of motivation. Moreover, the differences in results across EI levels underscore the significance of considering individual EI in understanding how burnout affects motivational processes and subsequent behaviors.

#### Burnout and the organizational dimension of OCB (OCB-O)

The mediational relationship of burnout through Autonomous Motivation and *OCB-O* (H9.2) was not supported, but the mediational role of Controlled Motivation for this relationship (H10.2) was partially supported for first-level and top managers and was fully supported for mid-level managers with high EI. These findings reflect on the subtleties in managerial levels, the importance of studying autonomous and controlled motivation forms, and the differences between OCB-I and OCB-O. For instance, for mid-level managers with high EI, the relationship between burnout and OCB-I is mediated by autonomous motivation, but controlled motivation mediates the relationship between burnout and OCB-O. The lack of mediation for Autonomous Motivation could suggest the relationship between burnout and OCB-O, unlike OCB-I, operates through more external sources of motivation than internal ones.

#### Burnout and the interpersonal dimension of WMB (WMB-I)

The mediational relationship of burnout through Autonomous Motivation and *WMB-I* (H9.3) was not supported, but the mediational role of Controlled Motivation for this relationship (H10.3) was partially supported for top managers and mid-level managers with high EI. These findings suggest that malfunctioning in terms of psychological health can lead to behavioral malfunctioning toward colleagues and supervisors through its impact on controlled motivation. The lack of support for Autonomous Motivation’s mediating role and partial support for Controlled Motivation could imply that external sources of motivation influence more strongly voluntary behaviors toward colleagues than internal ones when burnout is higher.

#### Burnout and the organizational dimension of WMB (WMB-O)

The mediational relationship of burnout through Autonomous Motivation (H9.4) and Controlled Motivation (H10.4) with *WMB-O* was partially supported at all managerial levels. These findings suggest a crucial relationship between burnout and work misbehavior in organizations. Burnout can lead managers to adopt destructive behaviors towards the organization through its impact on their autonomous and controlled motivation.

### Theoretical implications

The current study contributes to the literature in multiple ways. First, we have established the role of Burnout as a predictor, not just an end outcome, that can potentially decrease work motivation and affect other work-related behaviors. While the idea of Burnout as an independent factor is not new, it has been under-researched, and the findings emanating from the current paper support this claim. To the best of our knowledge, this is a pioneering paper in the OB/I-O Psych literature. As mentioned, the results support the notion of work burnout as an over-time process, meaning it can act as both an outcome of work-related constructs and a predictor of them (with exceptions, of course). These findings add to the literature on optimal functioning as they suggest burnout can impact work behavior indicators for both optimal functioning and malfunctioning.

Second, our research also joins the recent conversation in the literature that revolves around the negative aspects (“dark side”) of positively framed constructs (e.g., Davis and Nichols, 2016; Shkoler and Tziner, 2017; Wang and Lei, 2022; Shahzad et al., 2023). While high EI can facilitate the mediation of motivation between burnout and work behaviors, it can also have adverse implications. For example, in the case of the current study, high EI leads to an exacerbated relationship between burnout and motivation, such that the latter decreases considerably more, on account of being burnt-out, for individuals with high EI than those with low EI. Our findings communicate with Davis and Nichols’s (2016) call to examine the intricate interplay of when and why EI’s effects are detrimental and when they are beneficial.

Third, the current research provides insights into the subtle differences that distinguish managerial levels from one another, in terms of motivations, work attitudes, and behaviors at work (see also Rabenu et al., 2019). While most of the literature often focuses on nonsupervisory employees, our paper elaborates on the important and nuanced differences amongst managers (see also Giamos et al., 2023). Evidently, there are clear differences amongst the three managerial groups in our study; there are disparate behavioral and cognitive patterns, with first-level and top-level managers exhibiting similar patterns, with the mid-level managerial group showing a more dramatic difference in comparison to their counterparts.

Fourth, we support the dimensionality approach in research, using the constructs’ dimensions/facets as indicators without using the variable as a holistic latent factor. In our case, OCB and WMB were considered by their respective dimensions (i.e., interpersonal and organizational), which showed differentiating and interesting results. They are not predicted in the same manner by the same factors, which elucidates the need to perform more research that will use this approach, as it can help us reach a more nuanced and clearer understanding of organizational behavior phenomena.

Fifth, methodologically, it is paramount to investigate multivariate models in the work context. Reliance on simple bivariate analyses might lead to incorrect assumptions, biased results, and misleading implications. The results from the study also support the moderated mediation component of the research model, in which conditional factors (i.e., EI) significantly impact how mediational pathways operate. Our paper supports this notion, and we strongly recommend researchers to craft and design more elaborated research models that help fine-tune our understanding of human behavior in the workplace and explore a more accurate picture of the effects of job burnout.

### Practical implications

#### Prioritizing monitoring, recognition, and prevention of burnout

The present study underscores the significance of a process “from burnout to behavior,” illuminating that the repercussions of burnout extend beyond psychological and physiological states to impact behavior. Adopting this paradigm, practitioners can prioritize investment in employee well-being, recognizing its pivotal role in employee behavior and consequently, organizational performance. Amidst the pressures of productivity and competition, organizations and practitioners seek strategies to enhance employee behavior, but our research indicates that effective management of employee stress and burnout can facilitate this objective. Employees with improved well-being will contribute to the organization with their improved behavior. For instance, investment of resources (e.g., budget, time, attention) in burnout prevention and amelioration can in turn lead to performance and productivity improvements. This emphasizes the importance of constantly monitoring signs and symptoms of burnout and its early manifestations among managers to prevent burnout. Our findings point out the importance of pre-emptively recognizing indications of burnout among workers before it becomes severe or starts to impact the individual’s work motivation and behaviors significantly. For instance, HR practitioners may maintain timely or scheduled measuring of managers’ burnout and provide intervention and support in recognizing emotional exhaustion to prevent cynicism or inefficacy from developing because of mismanaged levels of burnout. Moreover, organizations can save financial costs due to burnout (e.g., absenteeism, turnover) by noticing its initial signs before they reach a “critical mass” point, addressing them proactively. This might involve observing changes in behavior, performance, and emotional well-being and actively listening to employees’ concerns and feedback (such as in hallway conversations or 1-on-1 with HR officials). Additionally, findings from the current study indicate that managing burnout early on, not waiting for it to continue developing further, would prevent loss of motivation, decrease workplace misbehaviors, and increase OCB.

#### Labor relations

Our findings indicate that burnout harms behavior, suggesting to policymakers that expected behaviors and conduct from employees depend on their level of burnout. While governments and legal bodies are increasingly introducing regulations such as minimum requirements for employment conditions and action plans to protect employees from stress and burnout, it does not guarantee a safe workplace for everyone. So, it becomes imperative to align expected employee conduct with their burnout levels, exposure to workplace stressors, and working conditions. For instance, the regulations related to dismissal for unsatisfactory performance or misconduct should consider the employee’s exposure to work stressors and its impact on their behavior.

#### Tailored motivation interventions

Our findings suggest that burnt-out managers show decreased work motivation at all managerial levels, and this association is contingent on the levels of their EI. This delineates that practitioners need to provide tailored motivational strategies for burnt-out managers. For instance, organizations may need to focus on the recognition and validation of burnout, reduction in workload (e.g., reassigning or reallocating responsibilities, adjusting deadlines), and support for burnt-out managers. Our findings regarding EI show that high EI increases (not decreases, as hypothesized) the negative impact of burnout on motivation. Thus, interventions to increase EI for burnt-out employees are not the surest way to increase motivation, as interventions to increase EI might result in a more profound decrease in motivation for burnout-out managers. As such, it is also crucial to tailor the motivation and/or EI interventions to the managerial levels. For instance, our results show that the association between burnout and motivation for “mid-level” managers with “low EI” is positive, but for “high EI,” it is quite negative. EI interventions for these managers should consider the evidence from our findings, suggesting that an increase in EI can lead to more awareness of burnout status that will impede motivation overall.

#### Recruitment and selection

In the context of recruitment and selection, it is prudent to conscientiously consider prospective managers’ EI levels. It is advisable to exercise caution in selecting individuals who demonstrate either excessively low or excessively high EI scores, as it is also recommended to “stay” at the mean or average level of EI (e.g., Davis and Nichols, 2016).

### Limitations and future research directions

The current study has several main limitations. First, we used single-source data. Such data rely on a single individual as a source of information; thus, our study is susceptible to self-report bias, as individuals may provide inaccurate or socially desirable responses. Furthermore, as we have used single-source data, we cannot cross-verify the data, limiting our ability to draw unbiased conclusions about our model. That said, the findings prove to be robust, intra-replicative, and not inflated in any way. Hence, future research should consider exploring our model with multiple sources/raters to provide a more accurate picture of how people respond to burnout. Furthermore, capitalizing on the recommendations and procedures provided in Conway and Lance (2010), the prevalence of a common method bias, even in a cross-sectional study, has minimal impact on the results, and, under certain conditions (e.g., moderation), it is mathematically irrelevant.

Second, data were also cross-sectional, which only allowed for capturing a “snapshot” of reality to test the research model at a single point in time. Hence, we could not explore temporal and trajectory between burnout, EI, OCB, and WMBs. Furthermore, cross-sectional data only allow us to study associative relationships between variables, as causal links are inappropriate and improper in this scenario. As such, future research should, for instance, consider establishing such links by using longitudinal panel data that could allow researchers to better capture these changes and establish temporal relations among constructs (Ployhart and Vandenberg, 2010), in addition to identifying how burnout unravels over time.

Third, the current study has limited external validity due to the cross-sectional nature of the data, and the solely Romanian sample, reflecting a very distinguished country and (work) culture. It is difficult to generalize the findings from the current research to other cultures or countries that have different national or work values (e.g., China or Australia) since each culture/country is unique (Hofstede, 1980, 1991). Thus, future research may focus on replicating the current findings in different cultural settings. For example, it could be interesting to examine cross-cultural differences in terms of how people from different cultures respond to burnout or how the unexpected buffering effect of EI unfolds. This is important to explore because replicating constructs in different samples allows researchers to test their validity (James et al., 1982). This could be of value to organizations that manage staff in different countries and strengthen their cross-cultural management practices.

Fourth, another minor limitation of our study is the unequal distribution of research participants across managerial levels: 513 first-level managers, 220 mid-level managers, and 107 top-level managers. This discrepancy in group sizes could potentially impact the statistical credibility and power of our findings. However, it’s important to note that the proportions of managers in each group are relatively representative of the overall managerial population in Romania. This means that the larger number of first-level managers compared to mid-level managers, and subsequently top-level managers, is logical given the hierarchical structure of Romanian organizations. Therefore, while the unequal sample sizes may introduce a minor bias, it can be considered marginal as it aligns with the natural distribution of managerial roles within the country.

Fifth, to reduce the complexity, we grouped motivation regulations into two higher-order factors of autonomous and controlled motivation. Recent findings suggest that this conceptualization may not be the most appropriate way of assessing work motivation as different types of controlled regulations relate differently to indicators of optimal functioning (Van den Broeck et al., 2021; Trépanier et al., 2023). It is recommended for future studies interested in differentiating motivation types to study them separately rather than grouping them into two overarching types (e.g., autonomous vs. controlled) to explain nuances in the relationship between lower-level types of motivation (e.g., introjected regulation) and optimal functioning.

Finally, future research should consider burnout as a predictor, not just an outcome. Understanding the predictive potential of burnout can deepen our understanding of the phenomenon and broaden our understanding of the outcomes of burnout and the more intricate processes that it is involved in (e.g., motivation). Such research is necessary for a deeper understanding of malfunctioning and plausibility of an antithesis for “happy-productive workers.” More research is needed to investigate the effect of burnout on other behavioral or attitudinal dimensions of functioning and their mediating mechanisms. For instance, it is relevant to investigate whether burnout can lead to lower performance vs. the reverse. This investigation is of utmost importance for the healthcare industry where the performance of workers leads to low-quality patient care and clinical errors (Trépanier et al., 2015). Future studies on burnout need to consider its process and changing nature, its development over time, and its reciprocal relationships with other work-related constructs (e.g., team cohesion, and quality of care for healthcare professionals).

## Conclusion

The current study provides valuable insights into the intricate relationships among burnout, work motivation, emotional intelligence, and behavioral outcomes across different managerial levels. By examining burnout, motivation, and work behaviors within the framework of managerial hierarchies, we provide valuable insights into organizational research and practices regarding the optimal functioning of managers.

Firstly, our findings reveal that burnout not only acts as an outcome (as conventional wisdom suggests) but also as a potent predictor of work behaviors. Specifically, we demonstrate that as burnout increases, autonomous and controlled motivation decrease, which, depending on the managerial level, impacts OCBs and WMBs. This highlights the importance of prioritizing monitoring, recognition, and prevention of burnout in organizational settings (as a process of assessment of psychosocial risks). Practitioners can benefit from investing in employee well-being initiatives, as early intervention can mitigate the adverse effects of burnout on behavior and ultimately enhance organizational performance.

Moreover, our results unveil the unexpected dark side of emotional intelligence, as it exacerbates the negative association between burnout and motivation, suggesting that heightened emotional intelligence may amplify the adverse effects of burnout on work motivation. This underscores the need for tailored motivational strategies, particularly for burned out managers, and cautious consideration of EI levels in recruitment and selection processes.

Lastly, our research contributes to a deeper understanding of managerial differences in motivation and work behavior. By examining distinct managerial levels, we elucidate the variations in behavioral patterns and motivational processes. This calls for a more nuanced approach to designing interventions and organizational policies that account for the diverse needs and challenges of managers at different hierarchical levels.

## Data availability statement

The data analyzed in this study is subject to the following licenses/restrictions: NA. Requests to access these datasets should be directed to OS, or.shkoler@gmail.com. For transparency consideration, it is important to note that the current study relies on a dataset from a larger and broader project; the dataset has been used in previous different research.

## Ethics statement

Ethical approval was not required for the studies involving humans because Ethics statements and consent for participation are included in the manuscript. The studies were conducted in accordance with the local legislation and institutional requirements. The participants provided their written informed consent to participate in this study.

## Author contributions

SS: Conceptualization, Writing – original draft, Writing – review & editing, Investigation, Project administration. OS: Formal analysis, Methodology, Writing – original draft, Writing – review & editing, Conceptualization, Investigation, Software, Validation, Visualization. DG: Writing – original draft, Writing – review & editing, Investigation, Validation. DC: Supervision, Writing – review & editing. ChV: Supervision, Writing – review & editing. AT: Resources, Data curation, Writing – review & editing. CrV: Resources, Data curation, Writing – review & editing.

## Appendix A

Based on Table 3, these are the results for hypotheses H1–H6:

1. Burnout negatively associates with Autonomous motivation at the first-level (*r* = −0.41^***^), mid-level (*r* = −0.26^***^) and top-level managers (*r* = −0.46^***^); the more burnt-out the employee, the less (autonomous) motivated they will feel.
2. Burnout negatively associates with Controlled motivation only at the first-level (*r* = −0.11^**;^ the more burnt-out the employee, the less (controlled) motivated they will feel), but *not* at the mid-level (*r* = −0.04) and top-level managers (*r* = −0.10).
3. Autonomous motivation positively associates with OCB-I at the first-level (*r* = 0.10), but *not* at the mid-level (*r* = −0.04) and top-level managers (*r* = 0.08).
4. Autonomous motivation *negatively* associates with OCB-O at the first-level (*r* = −0.16^***^), mid-level (*r* = −0.33^***^) and top-level managers (*r* = −0.19^*^); the more (autonomous) motivated the employee, they are less inclined to engage in OCB-O.
5. Controlled motivation positively associates with OCB-I at the first-level (*r* = 0.39^***^), but *not* at the mid-level (*r* = 0.50^***^) and top-level managers (*r* = 0.31^**^); the more (autonomous) motivated the employee, they are more inclined to engage in OCB-I.
6. Controlled motivation positively associates with OCB-O only at the mid-level (*r* = 0.29^***;^ the more (controlled) motivated the employee, they are less inclined to engage in OCB-O), but *not* at the first-level (*r* = 0.08) and top-level managers (*r* = 0.16).
7. Autonomous motivation negatively associates with WMB-I at the first-level (*r* = −0.55^***^), mid-level (*r* = −0.56^***^) and top-level managers (*r* = −0.54^***^); the more (autonomous) motivated the employee, they are less inclined to engage in WMB-I.
8. Autonomous motivation negatively associates with WMB-O at the first-level (*r* = −0.56^***^), mid-level (*r* = −0.56^***^) and top-level managers (*r* = −0.70^***^); the more (autonomous) motivated the employee, they are less inclined to engage in WMB-O.
9. Controlled motivation negatively associates with WMB-I at the first-level (*r* = −0.26^***^) and mid-level (*r* = −0.14^*^); the more (controlled) motivated the employee, they are less inclined to engage in WMB-I. However, the relationship is *non-significant* at the top-level managers (*r* = −0.12).
10. Controlled motivation negatively associates with WMB-O at the first-level (*r* = −0.34^***^), mid-level (*r* = −0.20^**^) and top-level managers (*r* = −0.26^**^); the more (controlled) motivated the employee, they are less inclined to engage in WMB-O.

## Appendix B

Tables 5, 6 indicate that only several indirect effects were statistically significant. The findings are as follows:

1. *Autonomous* Motivation did not mediate the relationship between Burnout and OCB-O, for any managerial level;
2. *Controlled* Motivation did not mediate the relationship between Burnout and OCB-I, for any managerial level;
3. *Autonomous* Motivation *fully* mediates the relationship between Burnout and OCB-I, at every E.I. level, for *first*-level managers;
4. *Autonomous* Motivation *fully* mediates the relationship between Burnout and OCB-I, only at mean and high levels of E.I., for *mid*-level managers;
5. *Autonomous* Motivation did not mediate the relationship between Burnout and OCB-I, for *top*-level managers;
6. *Controlled* Motivation *partially* mediates the relationship between Burnout and OCB-O, at every E.I. level, for *first*- and *top*-level managers;
7. *Controlled* Motivation *fully* mediates the relationship between Burnout and OCB-O, only at high E.I. level, for *mid*-level managers;
8. *Autonomous* Motivation did not mediate the relationship between Burnout and WMB-I, for any managerial level;
9. *Controlled* Motivation did not mediate the relationship between Burnout and WMB-I, for *first*-level managers;
10. *Controlled* Motivation *partially* mediates the relationship between Burnout and WMB-I only at high E.I. level, for *mid*-level managers;
11. *Controlled* Motivation *partially* mediates the relationship between Burnout and WMB-I on every E.I. level, for *top*-level managers;
12. *Autonomous* Motivation *partially* mediates the relationship between Burnout and WMB-O, at every E.I. level, for *first*-level managers;
13. *Autonomous* Motivation *partially* mediates the relationship between Burnout and WMB-O, only at mean and high levels of E.I., for *mid*-level managers;
14. *Autonomous* Motivation *partially* mediates the relationship between Burnout and WMB-O, at every E.I. level, for *top*-level managers;
15. *Controlled* Motivation *partially* mediates the relationship between Burnout and WMB-O, at every E.I. level, for *first*-level managers;
16. *Controlled* Motivation *partially* mediates the relationship between Burnout and WMB-O, only at high levels of E.I., for *mid*-level managers;
17. *Controlled* Motivation *partially* mediates the relationship between Burnout and WMB-O, at every E.I. level, for *top*-level managers;
